# Five new species and three new subspecies of Erebidae and Noctuidae (Insecta, Lepidoptera) from Northwestern North America, with notes on *Chytolita* Grote (Erebidae) and *Hydraecia* Guenée (Noctuidae)

**DOI:** 10.3897/zookeys.264.4304

**Published:** 2013-02-06

**Authors:** Lars G. Crabo, Melanie Davis, Paul Hammond, Tomas Mustelin, Jon Shepard

**Affiliations:** 1 Corresponding author. Adjunct Faculty, Dept. of Entomology, Washington State University, Pullman, Washington, USA; 724-14 St., Bellingham, Washington 98225-6302 USA; 2 Western Washington University, Bellingham, Washington USA; 3 Research associate, Department of Zoology, Oregon State University, Corvallis, Oregon USA; 4 11904 Tallwood Ct., Potomac, MD 20854 USA; 5 Research Faculty, Dept. of Entomology, Washington State University; 6420 Barabanoff Rd., Nelson, BC V1L 6Y1, Canada

**Keywords:** Canada, United States, Alaska, CO1, mitochondrial DNA, barcodes

## Abstract

Several taxonomic issues in the moth families Erebidae and Noctuidae are addressed for Northwestern North America. *Drasteria parallela*Crabo & Mustelin and*Cycnia oregonensis tristis*Crabo in the Erebidae and *Eudryas brevipennis bonneville* Shepard & Crabo, *Resapamea diluvius* Crabo, *Resapamea angelika* Crabo, *Resapamea mammuthus* Crabo, *Fishia nigrescens* Hammond & Crabo, and *Xestia perquiritata orca* Crabo & Hammond in the Noctuidae are described as new. The following new synonyms are proposed: *Chytolita petrealis* Grote with *Herminea morbidalis* Guenée; *Gortyna columbia* Barnes & Benjamin and *Gortyna ximena* Barnes & Benjamin with *Gortyna obliqu*a Harvey; and *Hydroecia pallescens* Smith with *Hydroecia medialis* Smith. The type locality of *Gortyna intermedia* Barnes & Benjamin is restricted to Lundbreck, Municipality of Crowsnest Pass, Alberta, Canada.

## Introduction

A website devoted to the macromoths (excluding the Geometridae) of the Pacific Northwest was recently created by a team that includes the authors of the current paper. The Pacific Northwest (PNW) is defined as all of Idaho, Oregon, and Washington and the western part of Montana in the United States and all of British Columbia in Canada. This site, called Pacific Northwest Moths (http://pnwmoths.biol.wwu.edu/ ), has been available to the public since July 2012. The Internet format–as opposed to a book or journal article–lends itself to continuous updating of things such as additional species, range extensions and life histories. However, it is not an appropriate format for taxonomic work intended for a larger audience, nor does it satisfy the requirements for the description of new taxa.

We are aware of several undescribed species of Erebidae and Noctuidae in the Pacific Northwest (PNW). Three of these are described here so that they can be included on the website.

The thorough vetting of PNW moth collections that was part of the website work confirmed that three superficially distinctive populations of previously named moths are isolated segregates. These are sufficiently different to warrant recognition as subspecies, a taxonomic category that we reserve for populations that are both geographically isolated and differ significantly in appearance from other populations of their species. Three subspecies are named in this paper.

One of the new PNW species is in the genus *Resapamea* Varga & Ronkay (Noctuidae). This presents an opportunity to name two additional species in this genus even though they are from parts of western North America outside the PNW.

A large number of taxonomic changes in the superfamily Noctuoidea have recently been advanced in a new check list for North America north of Mexico (Lafontaine and Schmidt 2010) and in the first update to this list (Lafontaine and Schmidt 2011). Additional synonyms to those published by these authors were discovered during the website work, usually because a PNW species is known by more than one name. The new synonyms that are discussed in this paper are in the genera *Chytolita* Grote (Erebidae) and *Hydraecia* Guenée (Noctuidae).

The genus *Hydraecia* is represented by *Hydraecia perobliqua* Hampson and the *Hydraecia obliqua* species-group in the PNW. The number of species in the latter group is confusing to collectors, with seemingly more names than species. An attempt by the senior author to revise it in the late 1990s was never published because of difficulties in assigning species boundaries using standard morphological methods. More recent DNA data allows many of these issues to be resolved. The PNW species *Hydraecia obliqua* (Harvey) and *Hydraecia medialis* Smith are discussed and illustrated herein, and the type locality of the third North American species in the species-group, *Hydraecia intermedia* (Barnes & Benjamin), is restricted.

This paper is arranged in check list order following Lafontaine and Schmidt (2010).

## Materials and methods

Genitalia were prepared using standard methods for inflating the male vesica and female bursa (Lafontaine 2004). Terminology for wing markings and anatomy also follows Lafontaine (op. cit.) except in *Drasteria* Hübner (Erebidae) where they are modified from Metlevski and Zolnerowich (2009). The term ductus bursae is used herein for the entire length of the tube connecting the ostium bursae to the corpus bursae. Metlevski and Zolnerowich (op. cit.) divide this structure into two parts–the ductus bursae and antrum–restricting the use of ductus bursae to the anterior sclerotized portion. In *Resapamea*, a stout process extending dorsad from the base of the sacculus is herein named the basal saccular process. This structure was called the clavus by Zilli et al. (2005), a term that is potentially confusing because the term clavus has been used previously for a rod-like sensory organ with apical setae located adjacent to the base of the valve in some noctuid genera (Forbes 1954, Lafontaine 2004).

The sequence of the 658 base pair “barcode” region of the mitochondrial cytochrome oxidase *c* subunit 1 (hereafter called CO1 or barcode) were obtained from the Barcodes of Life initiative through the courtesy of Dr. J. Donald Lafontaine. Only pre-existing data were used during this study. The barcode sequences were compared using phylograms constructed using the Kimura-2-Parameter distance model as implemented on the BOLD Systems website (Ratnasingham and Hebert 2007).

### The following collection acronyms are used:

AMNH American Museum of Natural History, New York, New York, USA

BMNH The Natural History Museum, formerly the British Museum (Natural History), London, England

CNC Canadian National Collection of Insect, Arachnids, and Nematodes, Ottawa, Ontario, Canada

CSUC C. P. Gillette Arthropod Biodiversity Museum, Colorado State University, Fort Collins, Colorado, USA

GPC Gary Peters Collection, Ocean Shores, Washington, USA

JHS Jon Shepard Collection, Nelson, British Columbia, Canada

JTT Jim Troubridge Collection, Selkirk, Ontario, Canada

LGCC Lars Crabo Collection, Bellingham, Washington, USA

ODA Oregon Department of Agriculture Collection, Salem, Oregon, USA

OSAC Oregon State Arthropod Collection, Oregon State University, Corvallis, Oregon, USA

TMC Tomas Mustelin Collection, Potomac, MD, USA

USNM National Museum of Natural History (formerly United States National Museum), Washington, D.C., USA

WFBM W. F. Barr Museum, University of Idaho, Moscow, Idaho, USA

WSU M. T. James Entomological Collection, Washington State University, Pullman, Washington, USA

## Species accounts

### Family Erebidae Leach, [1815]. Subfamily Arctiinae Leach, [1815]. Tribe Arctiini Leach, [1815]

#### Cycnia oregonensis tristis

Crabo ssp. n.

http://species-id.net/wiki/Cycnia_oregonensis_tristis

Figs 1 , 32

##### Type material.

**Holotype** male, USA, Washington, Thurston County, [WA]DNR Rocky Prairie, 4.2 mi. N of Tenino at Plumb, 46.92°N, 122.85°W, 75 m., 20.VI.1998, L. G. Crabo leg./ Barcodes of Life #CNCNoctuoidea12243. CNC. **Paratypes** 8 males. **USA. Washington.** Thurston County: Plumb, N of Tenino, TNC Rocky Prairie, elev 50 m., 46.92°N, 122.85°W, 26.VI.1990, L. G. Crabo leg. (1 male); [same locality and collector], 24.VII.1996; Puget Trough prairie (1 male); Rocky Prairie, 12.VII.1982, Don Frechin leg. (1 male); [same location data and collector as holotype], 4.VI.1998 (1 male); 20.VI.1998 (1 male); 24.VII.1998 (1 male); WA DNR Mima Mounds State Natural Area, 46.907°N, 123.049°W, 240’ [73 m.], 4.VI.1998, L. G. Crabo leg., mounded prairie (1 male); Mima Prairie, Thurston County Glacial Heritage Site, 46.86°N, 123.04°W, 120’ [37 m.], 10.VII.1998, D. Grosboll leg., prairie (1 male). CNC, LGCC, WSU.

##### Etymology.

The name is from the Latin *tristis* meaning sad, a reference to the gray color of this moth and the weather in its western Washington distribution.

##### Diagnosis.

*Cycnia oregonensis tristis* is distinguished by the uniform medium gray color of both wings. The nominate subspecies of *Cycnia oregonensis* (Fig. 2), found elsewhere in North America, has lighter yellow-cream to grayish-cream forewings with lighter veins and nearly white hindwings. *Cycnia oregonensis tristis* resembles superficially *Euchaetes egle* (Drury), an eastern North American tiger moth that occurs west to the eastern Great Plains. These moths are easily distinguished by locality.

##### Description.

**Head** – Antenna of male moderately bipectinate with each branch covered with fine cilia, black, dorsal shaft covered with light-gray scales. Female unknown. Scape light to medium dark gray with slightly lighter underside. Eye rounded, smooth. Labial palp moderately short with short apical segment, covered by short flat scales except slightly longer at ventral base; basal half yellow and distal half dark gray. Head covered in hair-like yellow scales except inferior and lateral border of frons whitish gray. **Thorax** – Vestiture of simple hair-like scales, light to medium dark brownish gray, slightly browner than forewing color, anterior portion near head yellow. Prothoracic collar slightly lighter than central thorax, with a gradual transition to yellow on each side. Tegula covered by long hair-like scales, lighter cream than central thorax. Legs: femur of foreleg yellow with gray ventral surface; other femora and the tibiae of all legs slightly brownish gray, smoky gray dorsally and lighter gray ventrally, tibiae lacking spiniform setae; tarsi light tan gray with a slight ochre tint, with three rows of short spiniform setae on each segment. **Wings** – Forewing length: males 19–20 mm. Forewing nearly even slightly smoky brownish gray, slightly darker on distal third; most specimens with a thin pale streak in fold and approximately half of specimens with veins on distal wing, cubital vein, and 1A+2A similarly pale. Transverse lines and all spots absent. Fringe white with base gray like terminal wing. Dorsal hindwing slightly brownish gray, slightly darker than forewing; half of specimens with slightly paler veins similar to those on forewing, lacking lines and discal spot. Hindwing fringe white with gray base. **Abdomen** – Color light putty gray with dorsal half of segments I–VI dark orange yellow; with rows of half-round black spots on segments I–VII comprised of a single larger spot in the dorsal midline and smaller rounded spots on lateral abdomen adjacent to lighter venter; ventral segment VII with a bilobed protuberance covered by modified scales (likely for pheromones). **Male genitalia** – Uncus short, hook-like, evenly tapered to a point, flanked on each side by a large strongly sclerotized block-like process directed posterolaterally with dorsal surface smoothly convex and ventral aspect concave, covered dorsally by innumerable velvety setae. Valve simple, membranous and strap-like, 3 × as long as wide; sacculus modified into a large sclerotized thorn-like process approximately 0.6 × as long and wide as valve, with acute tip directed slightly posteromedially. Aedeagus 7 × as long as wide, mesially constricted, with a pointed narrow cylindrical process arising from ventral aspect of distal third just to left of midline, projecting posteriorly and 20° toward right, and curving slightly dorsad. Vesica approximately 2/3 × as long as aedeagus, curved 90° dorsad from the tip of the aedeagus, with a large elongate conical apical diverticulum directed anteriorly–producing appearance of entire vesica curving 180°–and bearing a small basal patch of short cornuti. **Female genitalia** – Unknown.

**Figures 1–20.**
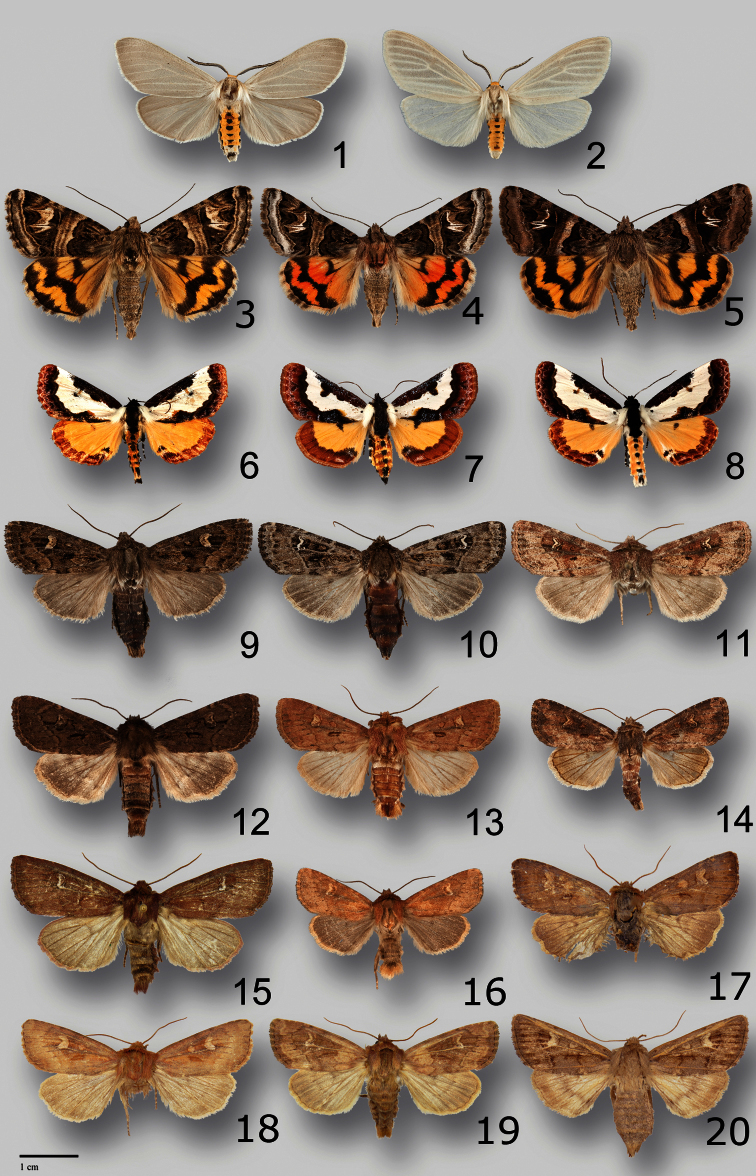
Adults of Erebidae and Noctuidae. **1** *Cycnia oregonensis tristis* Crabo, male paratype, WA, Thurston Co., Plumb **2** *Cycnia oregonensis oregonensis* (Stretch), male, Canada, BC, 5 mi W of Nelson **3** *Drasteria parallela* Crabo & Mustelin, male paratype, WA, Chelan Co., Chumstick Mountain **4** *Drasteria convergens* Mustelin, male, CA, Mono Co., Lee Vining **5** *Drasteria divergens* (Behr), male, WA, Garfield Co., FR40 at Sunset Point **6** *Eudryas brevipennis bonneville* Shepard & Crabo, female paratype, ID, [Gooding Co.], Wendell **7** *Eudryas brevipennis brevipennis* Stretch, male, USA, CA, [Riverside Co.], Riverside **8** *Eudryas unio* (Hübner), male, USA, MA, Norfolk Co., Ponkapoag Bog **9** *Resapamea diluvius* Crabo, male paratype, WA, Grant Co., Potholes **10** *Resapamea diluvius* Crabo, female paratype, WA, Grant Co., Potholes **11** *Resapamea diluvius* Crabo, female paratype, WA, Adams Co., Washtucna **12** *Resapamea passer* (Guenée), male, Canada, SK, 2 mi N Burstall **13** *Resapamea passer* (Guenée), male, USA, WA, Island Co., Joseph Whidbey State Park **14** *Resapamea passer* (Guenée), female, USA, Douglas Co., Badger Mountain **15** *Resapamea angelika* Crabo, male holotype, USA, NV, Elko Co., Angel Lake **16** *Resapamea innota* (Smith), male, USA, OR, Lake Co., Bull Prairie **17** *Resapamea mammuthus* Crabo, male holotype, Canada, YT, Old Crow **18** *Resapamea hedeni* (Graeser), male, Russia, Magadan Oblast, Tenkinsky District, Stokovyi **19** *Resapamea* species, possibly *Resapamea hedeni*, female, USA, AK, Unalakleet **20** *Resapamea* species, possibly *Resapamea mammuthus*, female, Canada, NWT, Aklavik.

##### Distribution and biology.

This subspecies is restricted to gravel prairies south of Puget Sound, Washington. These prairies were created by the outwash from the Vashon Lobe of the Pleistocene glaciation, and might have been maintained as open prairies by burning by native humans to promote the growth of camas lilies (*Camassia* spp., Liliaceae) as a food source. The moth is associated with Dogbane (*Apocynum* spp., Apocynaceae), the known foodplant of *Camassia oregonensis* elsewhere in North America (Tietz 1972). This is almost certainly the larval foodplant of *Camassia oregonensis tristis*, although this has not been confirmed. This moth flies during June and July.

##### Remarks.

*Cycnia oregonensis* is found in a large part of North America, occurring from coast to coast and from the border with Mexico north to central Saskatchewan and Nova Scotia (Covell 1984, Ferguson et al. 2000). This range includes much of the PNW, including western Oregon and the area east of the Cascade Range as far north as south-central British Columbia. Throughout most of its range it is nearly uniform in color and pattern. *Cycnia oregonensis tristis* is limited to a small area near Olympia, Washington and is the only known population of this species in Washington west of the Cascades. It is distinctly grayer and less patterned than all other populations, but has an identical CO1 barcode sequence.

The type specimen of *Euchaetes oregonensis* Stretch was collected by Lord Walsingham on a trip through Oregon during 1871–1872 (Stretch 1872–[1874]). Comparison of his itinerary (Essig 1941) with the flight period of the moth suggests strongly that it was collected in the southwestern corner of the state between Roseburg and the California border. *Cycnia oregonensis tristis* is separated from the closest west-side populations in western Oregon by 225 kilometers. All of the specimens of *Camassia oregonensis oregonensis* examined from near the likely type locality are similar to those from elsewhere in North America.

The Puget Prairies where *Camassia oregonensis tristis* flies are inhabited by several other distinctive Lepidoptera with limited distributions, including the noctuid moth *Apamea inordinata olympia* Crabo and several uncommon butterflies.

### Subfamily Herminiinae Leach, [1815]

#### Chytolita morbidalis

(Guenée)

http://species-id.net/wiki/Chytolita_morbidalis

Herminea morbidalis Guenée 1854: 56. Type locality: North America.
Chytolita petrealis Grote 1880: 219. Type locality: [USA], Ohio, Illinois, **syn. n.**
Chytolita fulicalis Smith 1907: 143. Type locality: [USA], Tennessee, **syn. rev.**
Zanclognatha punctiformis Smith 1895: 37. Type locality: [USA], District of Columbia, **syn. rev.**

##### Remarks.

Two species of *Chytolita* Grote have until now been recognized for North America (Lafontaine and Schmidt 2010): *Chytolita morbidalis* (Guenée) and *Chytolita petrealis* Grote. Both species names have at various times been used for material collected in the PNW; however, only a single species with slight variation in size and darkness exists in this region. Material from eastern North America, the type specimens of the available names, and material previously submitted for CO1 sequencing were therefore examined to attempt to elucidate the correct name for the PNW species. It was found that there is virtually no variation in barcodes in this genus despite the fact that the sample is diverse and includes larger light-colored specimens identified as *Chytolita morbidalis* and smaller dark ones submitted as *Chytolita petrealis* from a large portion of eastern North America. The two previously recognized species have been described as “identical in pattern” (Forbes 1954) and were only distinguished by size and darkness without structural differences. The dark small forms known as *Chytolita petrealis* are from swamps and acid bogs (Forbes op. cit.) and are consistent with an ecophenotype. This evidence indicates that *Chytolita petrealis* Grote is a synonym of *Herminea morbidalis* Guenée.

The two syntypes of *Herminea morbidalis* Guenée could not be located and might be lost; however, the original description is sufficient to identify the species and associate it with the genus *Chytolita* based on features of the labial palpus and the presence of an accessory cell on the forewing. A neotype designation is not necessary since there is only one *Chytolita* species in North America.

### Subfamily Erebinae Leach, [1815]. Tribe Melipotini Grote, 1895

#### Drasteria parallela

Crabo & Mustelin sp. n.

urn:lsid:zoobank.org:act:1FA8C27B-2159-41CA-BCF5-246F99B9F8B5

http://species-id.net/wiki/Drasteria_parallela

Figs 3 , 33 , 45

##### Type material.

**Holotype** male, USA, Washington, Chelan County: Chumstick Mtn, 47.63–.64°N, 120.44°W, 5100’ [1554 m.], 21.VI.2004, L. G. & E. K. Crabo leg. CNC. **Paratypes** 22 males, 2 females. **USA**. **Oregon.** Jackson County: Siskiyou Mts., Mt. Jackson summit and S slope on Rt. 20, 42.07–.08°N ,122.71–.72°W, 3.VIII.1995, Troubridge & Crabo leg., alpine and subalpine (1 male); Soda Mt. Rd., [42.065°, -122.478°], 28.VI.1986 (1 female); Klamath County: Keno, [1248 m.], 30.V.1939, S. Jewett, Jr. leg. (1 male); Lake County: Drake Pk., 6400’ [1951 m.], 29.VI.2006, H. E. Rice leg. (1 male); Warner Mts., S slope & summit of Drake Peak, 42.29°N, 120.14–.15°W, 7860–8220’ [2396–2505 m.], 2.VIII.1995, J. Troubridge & L. G. Crabo leg. (1 male). **Washington**. Chelan County: Chumstick Mtn, 47.63–.64°N, 120.44°W, 5100’ [1554 m.], 21.VI.2004, L. G. & E. K. Crabo leg. (6 males); Derby Cyn., 47.60–.62°N 120.49–.55°W, 2130–3130’ [649–954 m.], L. G. & E. K. Crabo leg. (1 male); Kittitas County, Quartz Mts., 47.074°N, 121.061°W, 1900 m., 7.VII.2005 (1 male); [same locality and collector], 14.VII.1990 (1 male); [same locality], 28.VII.2003, L. G. & E. K. Crabo leg. (1 male); Quartz Mtn., 6232’ [1900 m.], 1.VII.1998, K. Romain leg. (1 male); [same locality and collector], 7.VII.1998 (1 male); 28.VII.1998 (1 male); Klickitat County: Simcoe Butte, 45.99°N, 120.66–.71°W, 3240–4720’ [988–1439 m.], 7.VII.2000, L. G. & E. K. Crabo leg. (4 males); Yakima Co., Satus Cr., [46.217°, -120.433°], 28.V.1949, E. C. Johnston leg. (1 female); [same locality and collector], 29.V.1949 (1 male). CNC, LGCC, OSAC, TMC, WSU.

The type series is restricted to Oregon and Washington.

##### Etymology.

The name refers to the parallel lines across the pale medial area of the forewing of this species. This name perpetuates the geometry references of the related species *Drasteria divergens* (Behr) and *Drasteria convergens* Mustelin.

##### Diagnosis.

*Drasteria parallela* is most closely related to *Drasteria convergens* (Fig. 4), a species that occurs to the south of its range in California as far north as Mono County. Both have a medial line comprised of distinct parallel components, but the dorsal hindwing of *Drasteria convergens* is light red rather than orange. In the Pacific Northwest *Drasteria parallela* is most likely to be confused with *Drasteria divergens* (Fig. 5), which is similar in size and has a similarly colored orange hindwing. They can be distinguished without dissection by the pattern of the medial areas of both wings. The forewing medial line of *Drasteria parallela* is double with parallel component, whereas *Drasteria divergens* has a single broader medial line. On the hindwing, the discal spot of *Drasteria parallela* is nearly rectangular and the veins between it and the postmedial line are dark and contrasting. The discal spot of *Drasteria divergens* is joined broadly to the postmedial line giving it a long curved C-shape and the adjacent veins are orange. They also differ in other features of the maculation, including a smoother subterminal line in *Drasteria parallela*.

Structurally, the valves of *Drasteria parallela* and *Drasteria convergens* (Fig. 34) can be distinguished from those of *Drasteria divergens* by the nearly equal lengths of the two claspers and more rounded valvulae. In *Drasteria divergens* (Fig. 35) the right clasper is longer and more slender than the left and the posterodorsal valvulae are angular. *Drasteria parallela* has a relatively short and wide costal process of the right valve compared to that of *Drasteria convergens* in which it is long and narrow. In the vesica, the posteriorly-directed process arising from the glove-like diverticulum on the left side of the dorsal vesica is conical in *Drasteria parallela* and conical with two small finger-like projections in *Drasteria convergens*.

The female genitalia of *Drasteria parallela* and *Drasteria convergens* (Fig. 46) are told easily from those of *Drasteria divergens* (Fig. 47) by their much shorter antevaginal plates, which are rectangular and over half the length of the ductus bursae in *Drasteria divergens*, and by the presence of a narrow curved extensions of the pseudobursae that is absent in *Drasteria divergens*. The female genitalia of *Drasteria parallela* differ from those of *Drasteria convergens* by the size of the antrum of the ductus bursae, which is similar in length to the colliculus in *Drasteria parallela*, but twice as long in *Drasteria convergens*, and by the shape of the corpus bursae, which is rounded in *Drasteria parallela*, but ovoid in *Drasteria convergens* (ratio of width:length 0.87 and 0.7, respectively).

##### Description.

**Head** – Antenna of male filiform, densely ciliate ventrally with length of cilia approximately 1 × width of central shaft. Antenna of female filiform with single short cilia on sides of each segment. Scape covered in short tan scales. Eye round, smooth. Palpus covered in short gray-tan and scattered dark-gray scales, longer and lighter colored on ventral third. Frons smooth, it and top of head covered in long gray-tan scales. **Thorax** – Vestiture of long, narrow, apically notched, gray-tan and brown scales, forming vague tufts on posterior thorax. Prothoracic collar tan with longitudinal dark gray stripes at each side of head. Tegula covered in similar scales as thorax, with dark-brown medial and lateral stripes. Legs covered in gray-tan and scattered dark-gray scales; tibiae lacking spiniform setae; tarsal segments with three rows of spiniform setae. **Wings** – Forewing length: males 17–20 mm; females 18 mm (n=1). Forewing covered with brown, tan, and gray scales, ground color of wing base to antemedial line and subterminal areas dark brown with tan and lead-gray mottling, medial area light tan, darker near costa, and terminal area whitish gray to gray medially and blue gray to brown gray at margin, with a dark gray to black spot at apex; a complex mark resembling a large reniform spot in distal medial area between true reniform spot and strongly dentate postmedial line white medially and along dark crossing veins, tan laterally. Basal, antemedial, and postmedial lines similar, wide, partially double, black with chestnut-brown filling; basal line oblique from medial costa near wing base toward outer margin, fading at mid-wing; antemedial line complete, convex toward outer margin, drawn strongly toward wing base on costa, mid-wing, and posterior margin; postmedial line complex, costal origin near reniform spot, anterior portion strongly oblique toward outer margin to strong tooth on M1, slightly concave between M1 and M3, less strongly toothed on M3 and CuA1, then drawn strongly toward base and bending slightly anteriorly to posterior end of reniform spot, then bending sharply to meet posterior margin at a slight angle toward outer margin, portion posterior to reniform spot appearing to form a continuous line with medial margin of spot. Medial line dark gray, double ,with nearly parallel components of equal strength across width of wing. Subterminal line double with dark brown to black inner and gray outer components, filled with tan, smooth with slight undulations, slightly offset toward base below the costa and convex toward outer margin elsewhere, preceded by a black line or series of black wedges or smudged marks between veins. Terminal line black, slightly scalloped. Orbicular and claviform spots absent. Reniform spot ovoid or weakly C-shaped with wider posterior end, black, open posteriorly, filled with dark brown, much less conspicuous than pale medial area lateral to it. Fringe slightly scalloped, dark gray brown with tan to brown base, weakly checkered in a few specimens. Dorsal hindwing ground color dull light yellow orange to dull orange, with strong suffusion of gray scales at base and along inner margin and gray on veins from base to postmedial line; discal spot black, nearly trapezoidal, with wider posterior end and concave lateral margin, posterior portion not reaching or barely touching postmedial line; postmedial line black, slightly wider than discal spot, irregularly zigzag with three angulations, lateral on M2, medial on CuA1 and CuA2, and lateral between CuA2 and inner margin; terminal line black, slightly thinner than postmedial line with inner border roughly parallel to outer edge of postmedial line. Hindwing fringe similar to hindwing ground color, but with variable gray suffusion on and adjacent to veins, strongest near Rs, M1, and M3–CuA2. **Abdomen** – covered with a mixture of gray-tan and dark-gray flat and tan hairlike-scales, appearing even medium-dark gray brown. **Male genitalia** – Uncus strongly curved, slightly compressed laterally, with a dorsal ridge on distal half, tapering to acute apex. Scaphium long and narrow with articulation at uncus base. Juxta H-shaped, 1.2 × as high as wide. Valves racquet shaped, asymmetrical, right longer and wider than left (width:length 0.34 versus 0.26, respectively), with slight posterior projection from dorsolateral left valvula and rounded dorsolateral right valvula. Sacculus 4 × as long as wide, extending distally to mid-valve; saccular extensions asymmetrical, stronger on right with distal long tooth-like process projecting dorsomedially and weaker one on left with distal small point; pulvinus moderately strong on both sides. Costal processes roughly triangular, asymmetrical, 4 × as large on right as on left. Claspers symmetrical in length, 0.25 × length of valve, cylindrical, distal portions slightly asymmetrical with stronger curve toward midline on left than right; base of clasper raised into small rod-like cone with patch of fine setae at apex on both sides. Aedeagus tubular, 4 × as long as wide, with membranous granulose patch at dorsal left apex extending onto adjacent vesica base and sclerotized narrow extension onto ventral left vesica base (ventral plate of carina). Vesica bulbous, approximately 1 × as long as aedeagus and 1.2 × as wide as long, larger on left, with complex anatomy of multiple diverticula; ventral diverticula from left to right: mid-left-lateral spherical with granulose surface, basilar-left-lateral triangular with pointed anterior projection and granulose surface, ventral-basilar ovoid with two short projections to left, and mid-right-lateral spherical with granulose surface; dorsal diverticula from left to right: distal-left-lateral glove-shaped with seven small radial projections and single larger posterior projection at base, dorsal-distal ovoid with posteriorly-directed apex, and distal-right-lateral glove-shaped with four finger-like projections. **Female genitalia** – Ovipositor strongly telescopic. Ovipositor lobe long and narrow, 5 × as long as wide, tapering to a rounded point, covered sparsely by long hair-like setae that are most dense at apex. Abdominal segment VIII slightly longer than wide, covered by hair-like setae that are most dense at posterior margin; anterior apophysis 1.25 × as long as segment VIII and posterior apophysis 1.8 × as long as segment VIII. Ostium bursae nearly as wide as segment VIII, membranous ventrally, with sclerotized, broadly V-shaped, lamina postvaginalis posterior to ostium. Ductus bursae 1.5 × as long as segment VIII, nearly straight except for slight rightward bend at anterior end; antrum at posterior end short, similar in width to ostium and 1× as wide as long; lamella antevaginalis weak, 0.5 × as long as wide; strongly sclerotized tubular colliculum 1.8 × as long as wide, slightly longer on left side. Corpus bursae slightly asymetrically ovoid, nearly round, 1.15 × as long as wide, with small pseudobursa at right posterior end with narrow tubular extension from posterior end curving dorsad, anterior, and to right to project toward anterior right with ductus seminalis at tip.

##### Distribution and biology.

*Drasteria parallela* is found in the Cascade Mountains of Washington, the Klamath and Siskiyou Mountains of southwestern Oregon and northern California, and the northern Sierra Nevada in California. It is most commonly collected on exposed ridges in forests at middle elevations. It flies during July. The early stages and foodplant are unknown. Barcodes suggest a close relationship of *Drasteria parallela* and *Drasteria convergens* to *Drasteria howlandii* (Grote), which feeds on *Eriogonum* Michaux (Polygonaceae) (Powell and Opler 2009).

##### Remarks.

This species has until now been called *Drasteria convergens* in the PNW.

The barcode of a single sample of this species from Plumas County, California (BOLDSYSTEMS Sample ID: CNCNoctuoidea7767) differs by 1.3% from that of *Drasteria convergens* from Mono County, California.

### Family Noctuidae Latreille, 1809. Subfamily Agaristinae Herrich-Schäffer, [1858]

#### Eudryas brevipennis bonneville

Shepard & Crabo ssp. n.

http://species-id.net/wiki/Eudryas_brevipennis_bonneville

Figs 6 , 36 , 48

##### Type material.

**Holotype** male, USA, Idaho, [Twin Falls County], Twin Falls, 10.VII.1953, J. R. Douglass leg./ Database # CNC LEP 00094168/ Genitalia CNC slide #15232 male. CNC. **Paratypes** 5 males, 5 females. **USA. Idaho.** Gooding County: Wendell, 3500’ [1067 m.], 25.VII.1965, R. E. Miller leg. (1 female); Power County: Massacre Rocks S[tate] P[ark], 4290’ [1308 m.], 42.679°N, 112.987°W, 17.VII.2010, J. & S. Shepard leg. (1 male); Twin Falls County: Buhl, 3500’ [1067 m.], 29.VI.1961, R. E. Miller leg. (1 male); Kimberly, 1 mi. E., 10.VII.1970, A. C. Antonelli collector (1 female); Twin Falls [42.57°, -114.47°], 10.VII.1953, J. R. Douglas leg. (1 male); [same locality and collector], 7.VII.1945 (1 male), 9.VIII.1945 (1 female), 12.VII.1952 (1 female), [same locality and collector], 3700’ [1128 m.], 25.V.1953 (1 male); [same locality], 15.VI.1959, K. E. Gibson leg., Genitalia CNC slide #16535 (1 female). CNC, JHS, WFBM.

##### Etymology.

The species name is derived from Lake Bonneville. This glacial lake covered much of Utah and southern Idaho during the late Pleistocene epoch. The distribution of this moth is in the Lake Bonneville Basin and along its historic flood path along the Snake River.

##### Diagnosis.

No other Pacific Northwest noctuid is likely to be confused with this brightly colored moth. *Eudryas brevipennis bonneville*is superficially nearly identical to *Eudryas unio* (Hübner) (Fig. 8), which occurs in eastern North America as far west as the eastern Great Plains and Texas, but the genitalia of *Eudryas brevipennis bonneville* are indistinguishable from those of *Eudryas brevipennis brevipennis* Stretch from California. The long distal processes of the male aedeagus of *Eudryas brevipennis* (Fig. 36) are shorter by 20–25% compared to the lengths of the corresponding structures in *Eudryas unio* (Fig. 37). Similarly, the ductus bursae of females of *Eudryas brevipennis* (Fig. 48) is shorter thanthat of *Eudryas unio* (Fig. 49). The valves of *Eudryas brevipennis* (Fig. 36) are slightly shorter and less pointed than those of *Eudryas unio* (Fig. 37). In the females, the ductus bursae of *Eudryas brevipennis* is about 25% shorter than that of *Eudryas unio*, although these structures are difficult to measure precisely.

Subspecies *Eudryas brevipennis bonneville*differs from the nominate subspecies from California (Fig. 7) by the width of the dark marginal borders of both wings and darkness of the discal spots. The red-brown hindwing marginal band is relatively narrow and mottled in *Eudryas brevipennis bonneville***,** resembling those of *Eudryas unio*, whereas that of *Eudryas brevipennis brevipennis* is wider and uniformly darker. The reniform spot and hindwing discal spot are both black in *Eudryas brevipennis brevipennis*. Only the posterior part of the reniform spot is black in *Eudryas brevipennis bonneville* and its hindwing discal spot is smaller and fainter.

##### Description.

**Head** – Antenna of male nearly filiform with slight narrowing of the basal portion of each segment, covered with very short cilia on ventral portion. Antenna of female filiform similar, with longer cilia reduced in number to a single cilium on each side of each segment. Scape covered in short black scales, white ventrally. Eye round, smooth. Palpus covered in short black and occasional white scales, the latter most numerous on distal basal segment and near apex; middle segment relatively long. Frons with a conical central projection with slightly down-turned apex, covered in narrow black scales and a few white scales near base of antenna. Top of head covered with shiny black scales. **Thorax** – Vestiture centrally of shiny brown-black spatulate scales that form a loose posterior tuft, and laterally of hair-like white scales. Prothoracic collar covered centrally with shiny black scales, shorter anteriorly where they are mixed with a few white scales and longer posteriorly, and laterally with long pure white hair-like scales. Tegula covered entirely by long hair-like pure-white scales. Legs covered with shorter dark-gray, short white, and long white scales, longest on femur and tibia of foreleg and femora of mid-leg and hind-leg; with white rings on ends of segments; ventral tarsal segments with three rows of spiniform setae. **Wings** – Forewing length: males 15–16 mm; females 16–17 mm. Forewing ground color white with a pearly sheen; basal two-thirds of anterior wing from anterior cell to costal margin covered in dark-maroon, charcoal-gray, and a few lavender scales, darkest gray on costal margin and in distal cell and dark brown red in base of cell; middle third of posterior margin and subterminal area adjacent to postmedial line similar black, maroon, and lavender, widest on mid-wing to form a black and lavender patch bordered anteromedially by dark ochre; remainder of distal wing mottled red brown and lavender. Basal, antemedial and medial lines absent. Postmedial line thick olive brown, smoothly waved, positioned closer to outer margin than end of cell, roughly parallel to outer margin on anterior and mid-wing and more strongly curved basad near posterior margin to blend with posterior margin. Subterminal line powdery lavender, undulating, preceded by ill-defined patches of black, strongest opposite cell and between veins posterior to CuA1. Terminal line a series of red-brown lunules between veins. Orbicular spot blackish gray, elongate, fused to dark color of costa to form posterior margin of area in medial cell. Reniform spot diffuse, C-shaped, anterior portion blackish gray fused to dark costa like orbicular spot, mid-portion narrow, olive, and posterior end a diffuse black spot. Claviform spot absent. Fringe graybrown. Dorsal hindwing bright yellow. Hindwing discal spot dark gray to black, diffuse. Hindwing postmedial line reduced to a black spot at inner margin; marginal band circa 0.2× width of wing with undulating inner border, chestnut brown, darkest at anal angle and interrupted by yellow scales near veins; terminal line dark chestnut brown. Hindwing fringe red tan. **Abdomen** – Dorsal half bright yellow and venter whitish gray, with black spots on dorsal midline and each side of each segment and loose tufts of metallic brown-black scales on dorsal segments I–III. **Male genitalia** – Uncus rod-like, slightly curved, tapering gradually from base to apex with slight lateral widening near tip, distal portion slightly dorsoventrally flattened with a dorsal medial ridge, tapering to a downturned point. Anal tube sclerotized at base of dorsal portion and more extensively and strongly ventrally and laterally, ventral portion bulbous at base with ventral medial groove, tapering distally. Juxta bell shaped, 1.2 × as tall as wide, widest ventrally. Valve birdwing shaped; costal margin divided into two straight segments separated by a 45° downward bend at junction of proximal two-thirds and distal third, basal segment of costa sclerotized and angled 45° dorsad relative to attachment; posterior margin with bend dorsad at mid-point beyond sacculus, meeting costal margin at a point. Sacculus heavily sclerotized, extending to mid-point of valve, base 0.65 × as wide as valve tapering to 0.3 × as wide as valve near origin; modified rod-like clasper extending laterally from end of sacculus posterior and parallel to posterior margin of valve, tapering gradually from base to acute upturned tip. Distal valve lacking digitus, expanded cucullus, or corona, inner surface covered with innumerable fine setae distal to mid-portion. Aedeagus unusual, with very short basal segment divided at apex into two very long and narrow processes, longer distal portion identified as true aedeagus by small vesica at tip approximately 6× as long as base, arched strongly ventrad near base and becoming gradually straighter toward apex, total arc 180°; proximal process similar but shorter and more strongly arched, approximately 0.65 × as long as distal aedeagus, with slightly bulbous tip. Vesica membranous, minute. **Female genitalia** – Ovipositor lobes pad-like, covered sparsely with long and short fine setae. Abdominal segment VIII 0.75 × as long as wide; anterior apophysis 0.85 × as long as segment VIII, and posterior apophysis slightly shorter. Ostium bursae lightly sclerotized, ventral margin narrow and V-shaped. Ductus bursae sclerotized, very narrow, sinuous and crenulate, difficult to measure when mounted. Corpus bursae membranous, delicate, slightly ovoid (collapsed in figure), approximately 2.5 × as long as segment VIII, ductus seminalis joining posterior portion near junction with ductus bursae.

##### Distribution and biology.

*Eudryas brevipennis bonneville*occurs near large rivers and lakes in the northern Intermountain Region. Most specimens have been collected near the Snake River in south-central Idaho.

The moth flies during late spring and summer and has been collected from late May through early August. The early stages are unknown. California populations of *Eudryas brevipennis* feed on the willowherb *Epilobium ciliatum* Raf. and evening primroses (*Oenothera* spp.) (Comstock and Dammers 1938), all in the evening primrose family (Onagraceae). It is likely that *Eudryas brevipennis bonneville* utilizes similar plants in this family.

##### Remarks.

*Eudryas brevipennis*, including the Utah populations, is considered to be conspecific with *Eudrya unio* by Poole on the website Nearctica.com. These taxa differ in the structure of the genitalia of both sexes indicating that they are distinct species.

The identical male and female genitalia of the California and Intermountain populations of *Eudryas brevipennis* suggest strongly that they are the same species despite the differences in wing pattern and the 700 kilometer gap that separates them. A single specimen from Modesto, California in the CNC resembles subspecies *bonneville* more than other California populations. *Eudryas* should be sought in riparian habitats in the area between California and the range of *Eudryas brevipennis bonneville* to see if this subspecies has a larger range than is known currently. There is no barcode data for either subspecies of *Eudryas brevipennis*.

### Subfamily Noctuinae Latreille, 1809. Tribe Apameini Guenée, 1841

#### Resapamea diluvius

Crabo sp. n.

urn:lsid:zoobank.org:act:B5ED782B-3A1F-4404-92D8-DBEB0F415656

http://species-id.net/wiki/Resapamea_diluvius

Figs 9–11 , 38 , 50 , 53

##### Type material.

**Holotype** Male. USA, Washington, Grant County: Potholes, 1110’ [338 m.], 46.982°N, 119.451°W, 23.V.2001, L. G. & E. K. Crabo leg./ Database # CNC LEP 00094165. CNC. **Paratypes** 80 males, 25 females. **USA**. **Oregon**. Sherman County: Biggs, 1 VI 1960, S. G. Jewett (1 female); Biggs Junction, 28 IV 1959, S. G. Jewett (1 male). **Washington**. Adams County: Sand Hills near Washtucna, 9 mi. N. of Kahlotus, 46.78°N, 118.53°W, 445 m., 22.V.1999, L. G. Crabo leg., sand dunes (1 male, 1 female); Irrigation Exper. Sta. Basin unit [47.008°, -118.567°], 30.V.1963, E. C. Klostemeyer leg. (1 male); Franklin County: White Bluffs ferry, 46.675°N, 119.449°W, 25.IV.2002, Strenge/Zack leg. (1 male); Grant County: [same data as holotype] (30 males, 10 females); [same locality as holotype], 1095’ [334 m], 1.VI.2002, Crabo & Troubridge leg. (9 males, 2 females); [same locality and date as previous], Barcodes of Life CNCLEP70179 (1 male); stable dunes S of Moses Lake, 21.V.1994, J. Troubridge leg. (1 male); Road C SE at The Potholes, [same coordinates as holotype], 19.V.2001, J. Troubridge leg. (32 males, 9 females); [same locality and date as previous], Barcodes of Life CNCLEP70181 (1 female); Potholes, 1095’ [334 m.], 46.989°N, 119.425°W, 4.VI.2005, E. K. & L. G. Crabo leg. (1 male, 1 female); Vantage, 46°54'N, 119°56'W, 22.IV.1998, J. Troubridge leg. (2 males). CNC, CSUC, JHS, JTT, LGCC, OSAC, TMC, WSU.

##### Etymology.

The name is derived from the Latin *diluvium* meaning deluge or flood. The Columbia Basin where this moth occurs in Washington was scoured repeatedly by cataclysmic floods at the end of the Ice Age.

##### Diagnosis.

*Resapamea diluvius* is most likely to be confused with *Resapemea passer* (Guenée) (Figs 12–14), a common and widespread moth in North America. *Resapamea passer* is found in the Pacific Northwest where it flies on both sides of the Cascade Mountains, including at the type locality of *Resapamea diluvius*. *Resapamea diluvius* is less variable than *Resapamea passer*, which comes in a range in colors and patterns that includes dark brown, reddish brown, dull light yellow brown, or a mixture of light- and dark-brown forms. *Resapamea diluvius* is dark hoary gray brown or red brown, grayer than *Resapamea passer* on both wings, with a more streaky pattern distal to the cell due to pale-gray veins. A few specimens of *Resapamea passer* have streaky distal forewings, but this is due to dark veins against a lighter ground (the veins of most *Resapamea passer* are dark gray under magnification but do not contrast with the ground color). Most specimens of *Resapamea diluvius* have the streaky pattern accentuated by black between the veins in the distal subterminal area and terminal area across the width of the wing. In *Resapamea passer*, black scaling on the distal wing is uncommon, and is usually limited to the area distal to the cell and in the fold. Finally, white or cream in the lateral reniform spot is typical in *Resapamea diluvius*, whereas pale scales in the spot are variable in *Resapamea passer*: absent, darker yellow or tan if limited to the lateral portion, or filling the entire spot. Habitat association with dunes and early flight period are also characteristic of *Resapamea diluvius*, whereas *Resapamea passer* is found in a variety of wetland and agricultural habitats and usually flies later in the summer with a peak during late June and July.

The male genitalia of *Resapamea diluvius* are similar to those of *Resapamea passer* (Fig. 39). The cucullus of the valve is more massive than that of *Resapamea passer*, especially relative to the width of the valve, and the anal margin is more rounded. In the vesicas, the diverticulum bearing a ridge of spines (cock’s comb) on the ventral surface is positioned slightly closer to the base in *Resapamea diluvius*, is smaller in size, and has a less pronounced ridge of spines than in *Resapamea passer*.

The female genitalia of these species are also similar. The bursa copulatrix of *Resapamea diluvius* is rounder than that of *Resapamea passer* (Fig. 51), with the ratio of length to width approximately 1.5 in *Resapamea diluvius* and 1.75 in *Resapamea passer*.

##### Description.

**Head** – Antenna of male nearly filiform with a slight constriction at base of each segment, with dense ventral covering of short cilia. Antenna of female filiform with single cilia on each side of each segment. Scape light tan, with dorsal tuft of light-tipped brown to dark brown-gray scales. Eye round, smooth. Labial palp with lateral aspect of first two segments and entire short distal segment covered in short and flat gray-tan, gray-brown, and dark gray scales, elongating to a ventral fringe of brush-like dark gray-brown scales. Frons smooth, covered in narrow dark brown-gray scales. Top of head covered in relatively long and narrow light-tipped gray-tan to dark brown-gray scales. **Thorax** – Vestiture of collar, thorax, and tegula similar, a mixture of long, narrow, apically notched light-tipped dark gray-tan to gray-brown scales, appearing medium-dark to dark gray-brown with dusting of lighter scales on central tegulae. Legs even dark gray brown, with three rows of spiniform setae on basitarsus and four irregular rows on other tarsal segments. **Wings** – Forewing length: males 16–19 mm; females 15–18 mm. Forewing with a mixture of gray brown, gray tan, red brown, gray, and blackish-gray scales, ground color appearing medium-dark to dark gray brown or reddish-gray brown, usually slightly darker in cell and in medial area distal to reniform spot and often also posterior to cubital vein from base to postmedial line and in terminal area; costa anterior to radial vein hoarier gray; cubital vein and distal branches lighter gray-tan or gray, often with black suffusion between veins in medial area distal to reniform spot and across width of wing in distal subterminal and terminal areas. Basal and antemedial lines similar, gray, faint and incomplete, partially double filled with ground or a slightly lighter shade thereof; basal line evident as patches of gray scales on costa and near base of cubital line; antemedial line usually evident as a faint gray spot on costa and a strongly zigzag gray line posterior to cubital vein. Medial line gray to dark gray, diffuse, gently excurved, variable in prominence from absent, a faint shade on mid-wing, or complete. Postmedial line double with consistent medial black component and variable absent to faint-gray lateral component, filled with a light shade of ground color, drawn sharply basad to level of mid-reniform spot on costa, smoothly excurved around reniform spot with lateral apex on R5 or M1, then oblique and slightly concave toward base from near subterminal line to junction of basal two-thirds and distal third of posterior margin, very weakly toothed on veins except for a stronger tooth on 1A+2A. Subterminal line light gray tan, diffuse, slightly offset toward base near costa and undulating elsewhere, variable from a faint spot on costa to complete, preceded by aforementioned black scaling in distal subterminal area. Terminal line a series of black chevrons on veins. Orbicular and reniform spots completely or partially outlined in black; orbicular spot variably absent, faint, or prominent, a small to medium-sized oval filled with color of adjacent wing; reniform spot moderate sized, weakly to strongly kidney shaped, filled with white to cream laterally, less prominently inferiorly, and in a few specimens as an incomplete row of medial scales, ground color medially, and with a central dark-gray to black lunule that is strongest at posterior end. Claviform spot black, strongest anteriorly, narrow, small to moderate sized, filled with ground color or a darker gray shade thereof. Fringe ground color, usually with a lighter base and gray medial line. Dorsal hindwing slightly brownish gray, darker and grayer on distal half, with a gray ill-defined oval discal spot and thin terminal line. Hindwing fringe light gray brown with a gray medial line. **Abdomen** – dark brown gray. **Male genitalia** – Uncus cylindrical, evenly downcurved, distal portion tapering to a fine point. Tegumen with large penicillus lobes. Juxta shield shaped, 0.75 × as high as wide, with shallow V-shaped ventral margin. Valve S-shaped, 4.5 × as long as wide (measured at mid-portion), nearly even in width except for slight constriction at base of cucullus; with stout knob-like basal saccular process extending dorsolaterally from base and nearly reaching base of costa, medial margin of this process with triangular mesial projection at base and concave mid-section, apex rounded. Sacculus reaching to or slightly dorsal to costal margin and extending distally to mid-valve. Clasper a smooth ridge. Ampulla short, nearly spherical. Digitus a weak ridge, partially covered by medial edge of cucullus. Cucullus well developed with rounded apical and anal ends, 1.75 × as wide as mid-valve; mesial surface covered by fine setae; corona an irregular row of stout curved setae, partially double at anterior end. Aedeagus tubular, 4.5 × as long as wide, with a slightly elevated patch of minute spinules on ventral distal end. Vesica 0.67 × as long as aedeagus, bent 135° toward right at base to project anteriorly and toward right, basal half bulbous and distal half tubular, adorned with several diverticula and cornuti; a broad diverticulum on subbasal ventral surface with sclerotized apex with a cocks-comb row of short spines oriented along aedeagus axis and projecting ventrad; a membranous conical apical diverticulum on posterior vesica projecting anteriorly and rightward; a solitary cornutus on medial posterior surface comprised of a proximally-angled spine arising from a button-like base; and subapically, a patch of variable-sized spine-like cornuti directed proximally. **Female genitalia** – Ovipositor lobe elongate, dorsoventrally flattened and curved slightly ventrad at apex, 0.33 × as wide as long at base, narrowing near base to 0.25 × length, and tapering over distal third to a blunt point; proximal segments fused ventrally and dorsally to past mid-point; covered entirely but sparsely by long fine hair-like setae. Abdominal segment VIII 2 × as wide as long, anterior apophysis 1.25 × length of segment VIII and posterior apophysis 2.25 × length of segment VIII. Ostium bursae moderately sclerotized, ventral lip smoothly rounded and projecting posteriorly to cover ostium. Ductus bursae cylindrical, rugose, 1× as long as segment VIII and 1.33 × as long as wide. Corpus bursae unisaccate, broadly ovoid, 1.50–1.56 × as long as wide, slightly asymmetrical with rounded projection on left extending slightly posterior to attachment of ductus bursae with ductus seminalis at apex.

##### Distribution and biology.

This species occurs in the Columbia Basin in Washington and northern Oregon. Specimens from dunes in northern Nevada and the northern Great Plains have been examined but the limits of its distribution are not well known.

The early stages of *Resapamea diluvius* are unknown. The larvae most likely feed on *Rumex venosus* Pursh (Polygonaceae) based on their close association with this plant in the Columbia Basin. This rue is abundant on the dunes where *Resapamea diluvius* occurs (Fig. 53) and the moth has not been found in Columbia Basin dune systems from which the plant is absent. Some other *Resapamea* species such as *Resapamea passer* are known to feed on *Rumex* species (Tietz 1972*)*.

Adults fly from late April to early June. No specimens have been found at the type locality during summer or fall, although *Resapamea passer* occurs there during the summer. *Resapamea diluvius* is local but is often abundant where it occurs.

##### Remarks.

The CO1 sequences of *Resapamea diluvius* and *Resapamea passer* are nearly uniform within each species but differ from each other by over 3.5%.

*Hadena hulstii* Grote, *Hadena morna* Strecker, and *Hadena virguncula* Smith are considered to be synonyms of *Mamestra passer* Guenée (Lafontaine and Schmidt 2010). All were described from Colorado. The holotype of *Hadena hulstii* could not be located and is presumed lost (Lafontaine JD pers. comm. 2012). The holotypes of the other two were examined (*Hadena virguncula* from a photograph) to exclude the possibility that they refer to *Resapamea diluvius*.

#### Resapamea angelika

Crabo sp. n.

urn:lsid:zoobank.org:act:0F672160-B1C9-4A79-AEDC-0A01C47F6F0D

http://species-id.net/wiki/Resapamea_angelika

Figs 15 , 40

##### Type material.

**Holotype** Male. USA, Nevada, Elko County, Angel Lake (S side), SW of Wells, light trap, 1–2.VIII.2003, James K. & Eleaner Adams leg./ Database # CNC LEP 00094166. CNC. **Paratype** Female. USA, Nevada, Elko County, Rt 231, 11 mi SW Wells [Angel Lake], 23 July 2001, J. Troubridge/CNCNoctuoidea6305. JTT.

##### Etymology.

The name is derived from the type locality at Angel Lake, Nevada.

##### Diagnosis.

*Resapamea angelika* is a distinctive *Resapamea* species due to the combination of large size, even dark red-brown forewing color, small or absent orbicular spot, and narrow reniform spot with well-demarcated thin black outline and prominent cream to light orange filling. It is most likely to be confused with *Resapamea passer* (Figs 12–14) and *Resapamea innota* (Smith) (Fig. 16). *Resapamea passer* is variable, usually brown or mottled tan and brown, but is not dark red brown like *Resapamea angelika*. *Resapamea innota* has red-brown or orange-brown forewings and could potentially be confused with *Resapamea angelika* on this basis. Its orbicular spot is similar to the reniform spot, not very small or absent as in *Resapamea angelika*, and the reniform spot is broader, lacks a black outline and is filled with uniform light ochre, unlike that of *Resapamea angelika*. The dorsal hindwing of *Resapamea innota* is darker gray than in *Resapamea angelika*. *Resapamea angelika* appears to be a significantly larger moth (FW length 19 mm) compared to *Resapamea innota* (FW length 14–17 mm) .

The male genitalia of *Resapamea angelika* are similar to those of *Resapamea passer* (Fig. 39). Based on the holotype, *Resapamea angelika* has a slightly broader distal uncus, a more V-shaped inferior juxta, and a larger and more angular basal saccular process. In the vesica, the ridge of spines with a sclerotized base (cock’s comb) adorning a ventral diverticulum is smaller than in *Resapamea passer*.

##### Description.

**Head** – Antenna of male nearly filiform with slight constriction at base of each segment, covered ventrally by short cilia. Antenna of female filiform. Eye round, naked. Labial palp covered by short flat scales on sides, elongating to form a brush-like ventral fringe. Frons smooth, covered by narrow red-brown scales. Top of head covered by long and narrow red-brown scales. **Thorax** – Vestiture of collar, thorax, and tegula similar, a mixture of long, narrow, apically notched red-brown-tipped tan and red-brown scales, appearing even dark red brown, weakly tufted. Legs even gray brown, with three rows of spiniform setae on basitarsus and four irregular rows on other tarsal segments. **Wings** – Forewing length: male 19 mm. Forewing with a mixture of red-brown and gray-brown scales, ground color appearing even dark red brown, minimally darker in basal, medial and terminal areas; veins brownish gray, especially along radial and cubital veins, but not contrasting. Basal line absent. Antemedial line single, dark red brown, faint, ill-defined and incomplete, most evident on costa, in cell, and in fold, slightly offset toward base on costa and nearly transversely-oriented elsewhere. Postmedial line similar but more diffuse, oriented at 45° angle to wing from near end of cell to posterior margin, obsolete near costa. Subterminal line absent, its position in paratype indicated by a faint dark spot on costa in distal subterminal area and in holotype by proximal margin of darker terminal area. Terminal line thin, black, slightly thicker between veins. Orbicular spots small, oval, very faint in holotype, partially outlined in thin black bordered internally by cream, and filled centrally with ground color in paratype. Reniform spot moderate sized, narrowly kidney shaped, posterior portion partially fused to cubital vein in holotype, extending slightly posterior to vein in paratype, partially outlined by a thin black line strongest medially and absent anteriorly, filled peripherally with light whitish cream to light orange and containing a central or medially-positioned ground-color lunule. Claviform spot small, absent in holotype specimen and a small black smudge in paratype. Fringe brown gray with gray-orange base and darker gray medial line. Dorsal hindwing light gray orange to light fuscous, minimally darker and grayer on distal half, with slightly darker gray ill-defined narrow discal spot, veins, and terminal line. Hindwing fringe pinkish red brown with a light yellow-tan base. **Abdomen** – medium-dark brown-gray with slight red tint, especially posteriorly. **Male genitalia** – Uncus cylindrical at base, evenly downcurved, distal portion slightly dorsoventrally flattened and tapering to a fine point. Tegumen with large penicillus lobes. Juxta shield shaped, 0.5 × as high as wide, with V-shaped ventral margin. Valve weakly S-shaped, 5 × as long as wide (measured at mid-portion), tapering slightly from base to neck of cucullus; clavus large, reaching base of costa, rectangular with concave medial contour and angular apex. Sacculus reaching two-thirds of distance toward costa and extending distally to mid-valve. Clasper a smooth ridge. Ampulla short, nearly spherical. Digitus a weak ridge with a weak triangular projection, partially covered by medial cucullus. Cucullus well developed with rounded apical and slightly pointed anal ends, 1.65 × as wide as mid-valve; mesial surface covered by fine setae; corona an irregular row of stout curved setae, row partially double on dorsal half. Aedeagus tubular, 4.5 × as long as wide, with granulose patch on ventral aspect at base of vesica. Vesica 0.65 × as long as aedeagus, bent 135° toward right at base to project anteriorly and toward right, basal half bulbous, distal half tubular, with several diverticula and cornuti: weakly bulging subbasal diverticulum on ventral surface with sclerotized apex with a cock’s-comb row of short spines oriented along aedeagus axis and projecting ventrad; a membranous conical apical diverticulum on ventral vesica projecting anteriorly and toward right; a medial proximally-angled spike-like cornutus arising from a button-like base; and a subapical patch of short variable-sized spine-like cornuti directed basad. **Female genitalia** – Not available.

##### Distribution and biology.

This species is only known from the vicinity of Angel Lake in the East Humboldt Range of northeastern Nevada. The habitat is sedge meadows along tributaries of Angel Creek. These meadows appear to lack *Rumex* but harbor dense stands of an iris (*Iris* spp., Iridaceae) which might be the larval foodplant. The early stages of *Resapamea angelika* are unknown. The few known specimens have been collected during late July and early August.

##### Remarks.

The CO1 sequence of the female paratype differs from those of all other North American *Resapamea*, including *Resapamea passer* and *Resapamea innota*,by over 3.4%.

The holotypes of *Luperina innota* Smith, type locality Wyoming, Yellowstone Park, and *Luperina enargia* Barnes & Benjamin, type locality California, Tulare County, Monachee Meadows, were examined from photographs to ensure that neither name is referable to *Resapamea angelika*. These specimens are superficially very similar to each other and may represent the same species. Somewhat variable *Resapamea* populations resembling these types are found at mid-elevations in a large portion of the western United States and require further study to determine the number of species that are involved. Because of this, we feel that it is premature to consider *Resapamea innota* and *Resapamea enargia* to be synonyms.

#### Resapamea mammuthus

Crabo sp. n.

urn:lsid:zoobank.org:act:5D5927C7-717D-4DC4-B855-37389C705002

http://species-id.net/wiki/Resapamea_mammuthus

Figs 17 , 41

##### Type material.

**Holotype** Male. Canada, Yukon Territory, Old Crow, 5.VII.1983, R. J. Cannings leg./ Malaise trap. Forest edge on S-facing bluff/ Database CNC LEP 0094163/ SLIDE *Luperina* male ER8824. CNC. **Paratypes** None.

##### Etymology.

The name is derived from the genus of the wooly mammoth– *Mammuthus*. It is befitting of the moth because its Beringian distribution and relatively large size for the genus. It is a noun in apposition.

##### Diagnosis.

*Resapamea mammuthus* is unlikely to be confused with most other species of *Resapamea* in North America due to its northerly distribution and orange-tan color. It is superficially similar to *Resapamea hedeni* (Graeser) (Fig. 18), which occurs in Asia and might also occur in Alaska (see Remarks, below). The male genitalia of these species differ in the shape of the distal uncus. It is truncated in *Resapamea mammuthus* with a small T-shaped expansion at the tip (Fig. 41 inset) and tapered to a point in *Resapamea hedeni* (Fig. 42) as well as in all other North American *Resapamea* species. The vesica of *Resapamea mammuthus* differs from those of all other *Resapamea* discussed in this paper in lacking the subbasal diverticulum and medial cornuti. It differs from that of *Resapamea hedeni* in lacking a subbasal serrate (cock’s comb) cornutus.

##### Description.

**Head** – Antenna of male nearly filiform, with slight constriction at base of each segment, covered ventrally by short fine cilia. Antenna of female unknown. Scape orange tan, with dorsal tuft. Eye rounded, smooth. Labial palp covered laterally by short flat tan scales, lengthening to a brush-like fringe on ventral surface of first two segments. Frons smooth, covered in narrow orange-tan scales. Top of head covered in long narrow orange-tan scales. **Thorax** – Vestiture of collar, thorax, and tegula long, narrow, apically notched orange-tan scales, appearing medium-dark orange-tan [central thorax of holotype partially mildewed]. Legs light tan; with three ventral rows of spiniform setae on basitarsus and four irregular rows on other tarsal segments. **Wings** – Forewing length: male 21.5 mm. Forewing with a mixture of tan, orange-tan, gray-tan, light-gray, brown-gray, and gray scales, appearing medium-dark orange tan, grayer near anterior and posterior margins and darker gray-brown in terminal area; veins near costa, distal to postmedial line, and near posterior margin gray but not strongly contrasting; an ill-defined dark mark in medial area distal to lower reniform spot. Basal, antemedial, and postmedial lines faint, ill-defined dark gray with adjacent light orange tan. Basal line only evident near costa. Antemedial line evident on costa and posterior to claviform spot, forming an oblique dark mark on costa and a zigzag line from claviform spot to posterior margin. Medial line absent. Postmedial line very faint, ill-defined, smooth, strongly oblique toward base anterior to reniform spot, straight and parallel to outer margin lateral to spot, and slightly angled and concave toward base below spot to meet posterior margin at a right angle. Subterminal line light orange tan, faint, undulating; preceded by a faint indistinct shade of dark gray that is strongest opposite cell and in fold. Terminal line thin, dark gray. Orbicular spot round, outlined by ill-defined faint gray and filled with light orange tan. Reniform spot moderately large, kidney shaped with strong lateral indent, dark gray along medial and lateral sides and open anteriorly and posteriorly, filled with cream, slightly grayer at posterior end. Claviform spot black, ill defined, strong anteriorly and weak posteriorly, narrow, filled with ground color. Fringe gray tan, with a lighter tan base and gray medial line. Hindwing light gray tan with gray suffusion, very faint postmedial line, marginal band, terminal line, veins, and chevron-shaped discal spot. Hindwing fringe slightly lighter than hindwing ground color. **Abdomen** – tan [abdomen of holotype mildewed]. **Male genitalia** – Uncus cylindrical at base, evenly downcurved, distal portion slightly dorsoventrally flattened and truncated at apex with small lateral projections to appear T-shaped (Fig. 41 inset). Tegumen with large penicillus lobes. Juxta shield shaped, 0.5 × as high as wide, with V-shaped ventral margin. Valve S-shaped, 5.5 × as long as wide (measured at mid-valve), widest at base and cucullus, mid-section 2/3 as wide as base and tapering slightly to narrow neck at base of cucullus; stout sclerotized knob-like basal saccular process extending dorsolaterally from base to just dorsal to costal attachment of valve, medial margin of this process irregular and apex rounded. Sacculus reaching 2/3 of distance to costal margin and extending distally to mid-valve. Clasper a smooth ridge. Ampulla short, round. Digitus a weak ridge, partially covered by medial cucullus. Cucullus well developed with rounded apical and anal ends, 2 × as wide as mid-valve; mesial surface covered by fine setae; corona of stout curved setae, dorsal half partially double. Aedeagus tubular, 3.6 × as long as wide, with short linear extension onto ventral vesica bearing a loose row of very small spines. Vesica 0.7 × as long as aedeagus, bent 135° toward right at base to project anteriorly and toward right, basal two-thirds bulbous and distal half tapering, with a single conical membranous diverticulum on anterior side of distal vesica projecting anteriorly and a subapical posterior patch of variable-sized spine-like cornuti directed basad. **Female genitalia** – Unknown.

##### Distribution and biology.

This species is known only from the type locality at Old Crow, Yukon Territory. The habitat is described as forest edge on a south-facing hillside on the specimen label. The holotype was collected during early July. The early stages are unknown.

##### Remarks.

Two unidentified *Resapamea* females in the CNC, one from Unalakleet, Alaska (Fig. 19) and the other from Reindeer Station, Aklavik, Northwest Territories (Fig. 20), resemble *Resapamea mammuthus* and *Resapamea hedeni*. We exclude them from the type series of *Resapamea mammuthus* because their identity is uncertain until either population can be associated with males or until females of the Old Crow population of *Resapamea mammuthus* are found. Their superficial appearances suggest that the Aklavik specimen is the female of *Resapamea mammuthus* and that the Unalakleet specimen is *Resapamea hedeni* or a closely related species.

#### Hydraecia obliqua

(Harvey)

http://species-id.net/wiki/Hydraecia_obliqua

Figs 21–23

Gortyna obliqua Harvey 1876: 53. Type locality: [USA], California, [Mendocino]. NOTE: *Gortyna obliqua* was described from a single specimen. Poole (1989) states that the holotype is in the BMNH. This appears to be incorrect because all Harvey type material, including that in the BMNH, was examined by Barnes and Benjamin (1924) who concluded that a specimen in the AMNH labeled “Mendocino, California/4410/No. 10703 Collection Hy. Edwards/*Apamea obliqua*. Harv.” is the type.
Gortyna ximena Barnes and Benjamin 1924: 160. Type locality: [USA], California, Truckee. **syn. n.**
Gortyna columbia Barnes and Benjamin 1924: 161. Type locality: [Canada], B[ritish] C[olumbia], Saanich District. **syn. n.**

##### Remarks.

More than a decade ago, prior to the availability of mitochondrial DNA, the senior author conducted a study of the genus *Hydraecia* Guenée focusing on the western North American species related to *Hydraecia obliqua* (Harvey), herein referred to as the *Hydraecia obliqua* species-group for convenience. These moths occur over a large area spanning the Pacific Coast to the Great Plains west to east and British Columbia, Alberta, and Saskatchewan to southern California, northeastern Arizona, and northern New Mexico north to south. This study included examination of all primary types, assembling over 600 specimens from most large institutional and many private North American collections, and examination of over 60 genitalia preparations. The conclusion was that *Hydraecia intermedia* (Barnes & Benjamin), known only from the holotype from Fort Calgary, is distinct but that all other populations in the species-group exhibit nearly continuous clinal variation in maculation and genitalia to form a circle of races (rasenkranz) from California, across Montana, and ending in the montane forests of the Southwest. This work was accepted for publication but was withdrawn because of the author’s concern that somewhat distinctive populations on the Pacific Coast and in the Southwest might be different species from the widespread intervening population but that the genitalia structure lacked differences sufficient for resolving the species.

Since then, barcodes have demonstrated a consistent 2% difference between populations from near the Pacific Coast (northern California, Washington, and British Columbia (n=6)) and those from farther inland (Colorado, Washington, Wyoming, Alberta, and British Columbia (n=7)) that along with slight differences in maculation suggest that they are best treated as distinct species. The western species is *Hydraecia obliqua* and the eastern one is *Hydraecia medialis* Smith.

**Figures 21–31.**
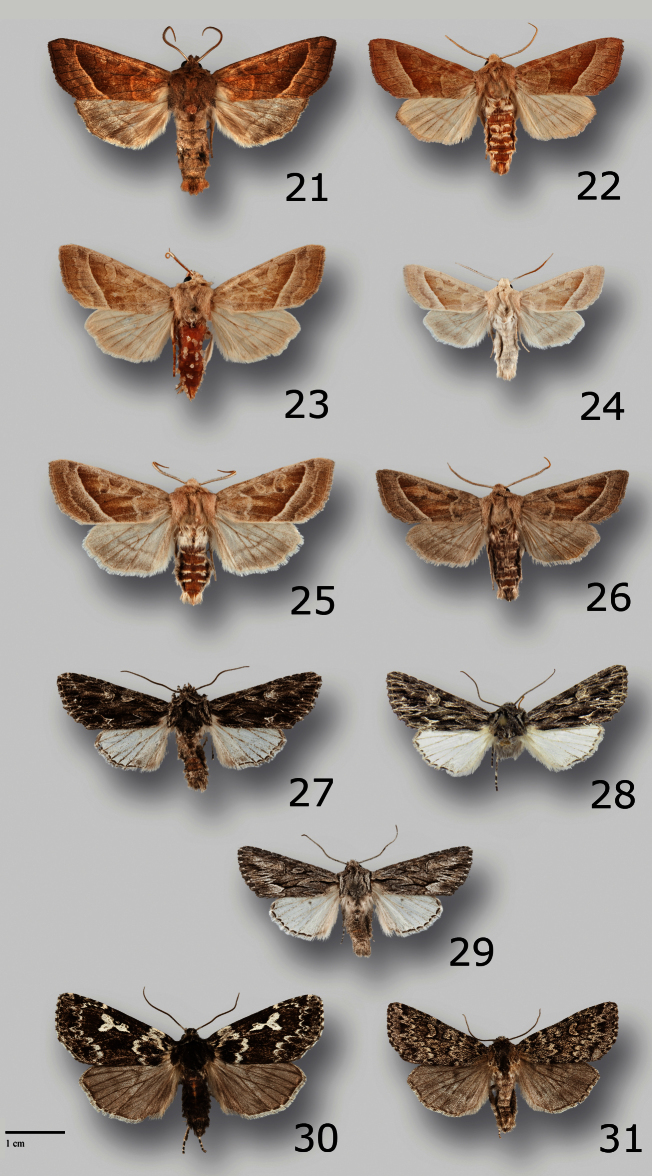
Adults of Noctuidae. **21** *Hydraecia obliqua* (Harvey), male, USA, CA, Sonoma Co., Bodega Bay Dunes **22** *Hydraecia obliqua* (Harvey), male, USA, WA, Island Co., Deception Pass State Park **23** *Hydraecia obliqua* (Harvey), male, USA, OR, Lane Co., S Fork McKenzie River near Cougar Reservoir **24** *Hydraecia medialis* Smith, male, USA, WA, Douglas Co., Jameson Lake **25** *Hydraecia medialis* Smith, male, USA, OR, Grant Co., South Fork John Day River **26** *Hydraecia medialis* Smith, male, Canada, BC, Princeton, Hayes Creek **27** *Fishia nigrescens* Hammond & Crabo, male paratype, USA, OR, Klamath Co., 6 mi. SE of Klamath Falls **28** *Fishia nigrescens* Hammond & Crabo, male holotype, USA, NV, Lander Co., 3 mi. W of Kingston **29** *Fishia yosemitae* (Grote), male, USA, OR, Lake Co., Deep Creek W of Adel **30** *Xestia perquiritata orca* Crabo & Hammond, male paratype, OR, [Lincoln Co.], Newport **31** *Xestia perquiritata partita* (McDunnough), male, WA, Pend Oreille Co., Salmo Mountain.

*Hydraecia obliqua* occurs east to the Sierra Nevada in California and the crest of the Cascade Range in Oregon and Washington. It occurs continuously on the coast north to southwestern British Columbia, with a disjunct northern population at Terrace, British Columbia. Its forewing is warm orange brown, varying considerably in darkness from dark brown on the California Coast (Fig. 21), lighter orange brown in the Pacific Northwest (Figs 22–23), and pale yellow brown in the Sierra Nevada. The hindwing is pale with a yellow tint, usually with dark veins and a gray suffusion in the submarginal area. The forewing pattern is similar to that of *Hydraecia medialis*, especially where they approach each other in the PNW, with slight differences that are described under *Hydraecia medialis*.

An area of possible intergradation between *Hydraecia obliqua* and *Hydraecia medialis* in Oregon is discussed under *Hydraecia medialis*.

**Figures 32–37.**
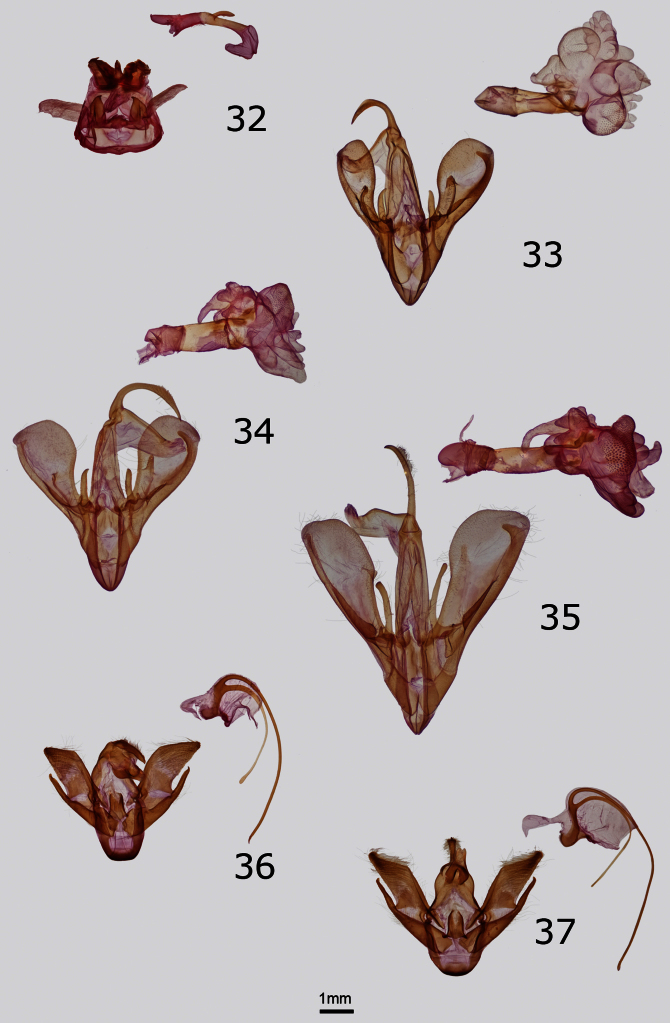
Male genitalia of Erebidae and Noctuidae. Ventral or right aspect of aedeagus is shown. **32** *Cycnia oregonensis tristis* Crabo, paratype, USA, WA, Thurston Co., Plumb (ventral aspect) **33** *Drasteria parallela* Crabo & Mustelin, paratype, USA, WA, Klickitat Co., Simcoe Butte **34** *Drasteria convergens* Mustelin, USA, CA, Mono Co., Lee Vining **35** *Drasteria divergens* (Behr), USA, OR, Baker Co., Burnt River Canyon **36** *Eudryas brevipennis bonneville* Shepard & Crabo, paratype, USA, ID, Twin Falls Co., Buhl **37** *Eudryas unio* (Hübner), USA, MA, Norfolk Co., Ponkapoag Bog.

#### Hydraecia medialis

Smith

http://species-id.net/wiki/Hydraecia_medialis

Figs 24–26

Hydroecia medialis Smith 1892: 251. Type locality: [USA], Colorado.
Hydroecia pallescens Smith 1899: 25. Type locality: [Canada], Alberta, Calgary. **syn. n.**

##### Remarks.

*Hydraecia medialis* is the widespread and variable species that occurs east of the range of *Hydraecia obliqua*. It is similar to it in size and pattern but is duller gray brown in the PNW, ranging from very pale (Fig. 24) to darker gray brown (Figs 25–26). The forewing postmedial line tends to be more angled relative to the posterior margin than in *Hydraecia obliqua* and usually lacks a slight bend in or near the fold that is found commonly in that species. The hindwing ground color is variable, most commonly off-white, but lacks a yellow tint. Gray scaling on the hindwing varies from absent to covering the entire wing. When present on the distal portion, it often forms a band to the outer margin, whereas in *Hydraecia obliqua* gray shading usually leaves the outer edge of the wing pale. Specimens of *Hydraecia medialis* from forests tend to be darker than those from open sage steppe habitats, especially in southern British Columbia and western Montana. Its northern limit in the PNW is at 100 Mile House in south-central British Columbia.

Specimens of *Hydraecia medialis* from the Great Plains are similar to those in the PNW but tend to be more uniform with dull gray-tan color, smooth lines, and less contrast between the antemedial and medial areas on the inner third of the forewing. Populations from the southern Rocky Mountains have a similar pattern to those from the Great Plains but are more colorful and variable, with gray or red-brown individuals and paler gray-white subterminal areas. Those from Utah and Arizona are red brown, darkest in Arizona.

As mentioned under *Hydraecia obliqua*, the barcodes of *Hydraecia obliqua* and *Hydraecia medialis* differ by 2%.A third barcode haplotype differing from both of these by slightly more than 2% exists for a single specimen of *Hydraecia medialis* from Wyoming (BOLDSYSTEMS Sample ID: CNCNoctuoidea6703). This specimen is superficially indistinguishable from two other Wyoming specimens with barcodes that match those of other *Hydraecia medialis* and this haplotype is therefore interpreted as a DNA polymorphism rather than evidence of a cryptic species.

A discussion of *Hydraecia intermedia*–known only from the holotype–is warranted in this section because its type locality of Fort Calgary suggests that it should be sympatric with *Hydraecia medialis* near present-day Calgary in southwestern Alberta. Its forewing is warm yellow brown unlike those of *Hydraecia medialis*, with markings that are more like those of *Hydraecia obliqua* than *Hydraecia medialis*. Structurally, its digitus is shorter and more bluntly rounded than those of all other populations in the *Hydraecia obliqua* species-group (n=45). The following discussion regarding its type locality is contributed by B. C. Schmidt:

“*Hydraecia intermedia* is an enigmatic taxon that has not been recorded near the stated type locality, nor anywhere else in Alberta (Pohl et al. 2010), since the collection of the type specimen about a century ago. In Barnes and McDunnough’s (1924) original description, the type specimen data is given as “Ft. Calgary, N. W. Brit. Columbia” and “VIII, 16” without a mention of year or collector. Virtually all of the moth specimens originating from the Calgary area in the early 1900’s were collected by the well-known pioneer lepidopterist Frederic Hova Wolley Dod who resided in the foothills just west of Calgary (Bird et al. 1995), and who was the source of many moths named by J. B. Smith (Todd 1982). However, Dod’s specimens were never labelled as “Ft. Calgary” nor “N. W. Brit. Columbia” – this convention appears to have been used solely by J. Gamble Geddes, who made extensive Lepidoptera collections in south-western Alberta during his visits in 1883 and 1884 (Geddes 1889). It is not clear why Geddes referred to the region as North West British Columbia, as the modern boundaries of British Columbia were already established at that time, while southern Alberta was known as the district of Alberta and was part of the North West Territories. A subsequent note on his collecting again shows that he referred to the region as British Columbia (Geddes 1889), possibly because he considered the mountains and passes he visited to be part of B.C. (the passes indeed straddling the Alberta – B.C. boundary). Several butterflies (*Lycaena dorcas florus* W. H. Edwards, *Colias elis* Strecker) and moths collected by Geddes during these trips were named as new species. Geddes’ handwritten catalogue of butterflies in the CNC Entomology Library indicates that Geddes collected butterflies in the Crowsnest Pass area in August of 1883, and was collecting in the Crowsnest Pass proper on August 16^th^, corresponding to the “VIII 16” of the *intermedia* holotype. As there was no direct rail line between the Crowsnest Pass and Calgary, some 350 km distant, it is very unlikely that the *intermedia* type could have been collected on the same day in Calgary, and it appears that Geddes simply recorded the nearest major settlement before distributing the specimens and associated label data. A week earlier, Geddes collected the type specimens of *Lycaena dorcas florus* (W. H. Edwards) at “Garnett’s Ranche” near Lundbreck at the mouth of the Crowsnest Pass (Bird and Ferris 1979). As Geddes visited and likely also stayed at Garnett’s Ranch, which served as a base for geology field parties (Inglis 1978), Geddes undoubtedly also collected moths at the ranch. The type locality of *Gortyna intermedia* Barnes & Benjamin is therefore restricted to Lundbreck, Municipality of Crowsnest Pass, Alberta. The diverse montane fauna of southwestern Alberta continues to yield previously undocumented moth species (Schmidt 2007), and the persistence of an undiscovered population of *Hydraecia intermedia* is certainly possible.”

The holotype of *Hydraecia intermedia* resembles the single specimen of *Hydraecia obliqua* in the CNC from Terrace in west-central British Columbia. This locality is far north of the continuous distribution of *Hydraecia obliqua*, which ends near Vancouver, British Columbia. This specimen is structurally similar to other *Hydraecia obliqua* populations, not *Hydraecia intermedia*. All British Columbia *Hydraecia obliqua* species-group specimens from east of the Cascade Mountains and British Columbia Coast Ranges are typical *Hydraecia medialis*, including from Cranbrook which is the closest locality to the Crowsnest Pass locality of *Hydraecia intermedia*. Nonetheless, it is interesting to speculate that *Hydraecia intermedia* could be an eastern population of *Hydraecia obliqua*. We retain *Hydraecia intermedia* as a species because of the structural differences between it and *Hydraecia obliqua*, and because there are no records of similar specimens in central British Columbia between Terrace and Crowsnest Pass.

Although the present reduction of this species-group to three species–*Hydraecia intermedia*, *Hydraecia medialis*, and *Hydraecia obliqua*–is best supported by the available data there are two remaining issues that cannot be solved with the information at hand. A large series of specimens from the east slope of the Cascades at Camp Sherman, Jefferson County, Oregon at OSAC show possible intergradation between *Hydraecia obliqua* and *Hydraecia medialis*, with some specimens that are difficult to assign to either species. This raises the possibility that the original hypothesis that *Hydraecia obliqua* and *Hydraecia medialis* are the same species could be correct despite the different barcodes in coastal and interior populations. Barcode or other DNA data from this population might help to elucidate its significance but is not available.

Similarly in the Southwest, at the other end of the rasenkranz, more DNA samples from Colorado, Utah, and Arizona would be helpful to exclude the presence of an undiscovered species amongst the colorful populations that occur there. Of these, a dusky red-brown population from east-central Arizona is the most distinctive.

### Tribe Xylenini Guenée, 1837. Subtribe Antitypina Forbes & Franclemont, 1954

#### Fishia nigrescens

Hammond & Crabo sp. n.

urn:lsid:zoobank.org:act:B23F75CA-E5EA-4891-AEFC-19C4DE6035CD

http://species-id.net/wiki/Fishia_nigrescens

Figs 27–28 , 43 , 52

##### Type material.

**Holotype** Male. [USA], Nevada, Lander Co., 3 mi W of Kingston, Kingston Cr., 7150’ [2179 m.], 30.IX.2000, Laurence L. Crabtree leg./Database for Noctuoidae [sic] 14832/Genitalia CNC #15215/Barcodes of Life Project, Leg removed, DNA extracted. CNC. **Paratypes** 7 males, 1 female. **USA**. **California.** Mono County: Dunes NE of Mono Lake, 23.IX.1995, R. Robertson leg. Barcodes of Life Noctuoidea 14834 (1 male); [same data as previous], Barcodes of Life Noctuoidea 14833 (1 male); Riverside County: Pinyon Crest, 4000’ [1219 m], 5.XI.1966, R. H. Leuschner leg. Genitalia slide CNC 15223 (1 male). **Oregon**. Deschutes County: Cline Falls State Park, J. C. Miller coll., Larva 25.V.1995 on *Chrysothamnus nauseosus*, Pupa 19.VI.1995, Adult 28.IX.1995 (1 male); Grant County: John Day Fossil Bed N. M., Sheep Rock Unit U. C., 2.X.2003, U.S. Natl. Park Service leg. (1 male); Jefferson County: Warmsprings, 27.X.[19]52, S. G. Jewett Jr. (1 female); Lake County: Hwy. 20 at Glass Butte, 23.X.2009 U.S.D.A (1 male); Klamath County: 6 mi. SE of Klamath Falls, 14.X.1964, Kenneth Goeden, Blk. Light trap (1 male). CNC, OSAC.

The type series is restricted to California, Nevada, and Oregon. Two additional specimens from Mt. Lemmon Highway, Pima County, Arizona at the CNC are excluded from the type series.

##### Etymology.

The name is derived from the Latin *niger* meaning black or dusky. It refers to the forewing color of the moth.

##### Diagnosis.

*Fishia nigrescens* is distinguished from other North American *Fishia* species by the charcoal-gray forewing without warm brown or reddish shades. Other western North American species are either much lighter gray (*Fishia yosemitae* (Grote) (Fig. 29)) or have brown color on the forewing (*Fishia discors* (Grote) and *Fishia connecta* (Smith)). The reniform spot of *Fishia nigrescens* is arrowhead shaped with a deep lateral indentation. That of *Fishia yosemitae* is an upright ovoid shape with only a weak lateral indentation.

In the male genitalia, the valve of *Fishia nigrescens* can be told from that of *Fishia yosemitae* by the shape of the digitus. In *Fishia nigrescens* the two prongs of the bifid digitus are unequal in length with a long dorsal and short ventral process. In *Fishia yosemitae* these processes are shorter and of similar length.

The female genitalia of *Fishia nigrescens* differ from those of *Fishia yosemitae* in the shape of the left posterior projection of the corpus bursae, blunter and more conical in *Fishia nigrescens* and rounder in *Fishia yosemitae*. The bursa of *Fishia nigrescens* has five signa whereas that of *Fishia yosemitae* has three, lacking two small signa at the anterior end.

##### Description.

**Head** – Antenna of male biserrate and fasciculate. Antenna of female filiform. Scape with anterodorsal tuft of long gray scales and an anteroventral tuft of white scales. Eye rounded, smooth. Labial palp covered laterally by a mixture of short flat tan and black scales, lengthening to a brush on ventral portion of first two segments; short distal segment covered in white and gray scales. Frons covered in short, narrow, bifurcate scales, light gray centrally and dark gray laterally. Top of head covered in longer white-tipped gray scales. **Thorax** – Vestiture of bifurcate and trifurcate gray, white, and white-tipped gray scales, appearing slightly hoary dark gray, with short paired anterior tufts behind collar. Prothoracic collar gray with a gull-wing-shaped black transverse line across mid-portion and a pale edge. Tegula gray with black lines parallel to medial and lateral margins. Legs gray with dark- and light-gray barring on tarsal segments; lateral tibia with partial loose row of spiniform setae; tarsal segments with three ventral rows of spiniform setae. **Wings** – Forewing length: males 19–20 mm; female 18 mm. Forewings of males and females similar. Forewing ground color slightly mottled charcoal gray, darkest in median area in fold and in subterminal and terminal areas opposite cell and in fold; lightest near apex and in mid-terminal area; distal veins black; basal dash thin, black; a thicker black line spans median area in fold, and a similar shorter line present anterior to M2 distal to reniform spot. Basal and antemedial lines similar, dark gray with lighter-gray filling. Basal line evident on costa and near cell, appearing broken. Antemedial line dentate, strongly on mid and posterior part of wing, with a long tooth toward lateral margin on 1A+2A. Medial line dark gray, faintly evident only on costa. Postmedial line two black spots on costa, faint and difficult to identify near end of cell, gray to black and filled with white on mid- and posterior part of wing, strongly serrate on mid-wing, forming a strong black and white tooth toward base in fold. Subterminal line pale gray, incomplete, with a series of white spots between veins on mid-wing; preceded by a series of long black chevrons between veins on mid-wing. Terminal line a series of black wedge-shaped spots between veins. Orbicular and reniform spots outlined incompletely by thin black line. Orbicular spot elongate, oval, filled with light gray with a central dark-gray line parallel to long axis. Reniform spot moderately large, broadly C-shaped with deep lateral indentation, posterior part extending farther laterally than anterior part, filled with light gray with a dark-gray line in medial and inferior portion. Claviform spot black, darkest posteriorly, inconspicuous due to dark adjacent ground. Fringe scalloped, dark gray with a medial black line, checkered with whitish gray at ends of veins. Dorsal hindwing white with a slight sheen in males and medium-dark gray in females, with dark gray veins, faint gray discal spot, and black terminal line in both sexes. Hindwing fringe white in male, light gray in female, with an incomplete gray medial line and scattered gray scales in basal row in both sexes. **Abdomen** – Abdomen covered with a mixture of white-tipped gray and white flat and gray hair-like scales, appearing powdery medium gray. **Male genitalia** – Uncus relatively short and nearly straight, pointed at tip. Tegumen with weak penicillus lobes. Juxta nearly rectangular, 1.4 × as long as wide, dorsal portion at opening for aedeagus slightly narrower than base. Valve elongate, 5.5 × as long as wide, widest at junction of proximal two-thirds and distal third near digitus, distal third angled 30° dorsad relative to base; sacculus fairly weak, tapering evenly from base to slightly beyond mid-valve; small ampulla present; clasper a sclerotized ridge; digitus prominent, cylindrical, projecting 45° ventrad and distal relative to basal axis of valve, with bifurcation at mid-point with long dorsal and short ventral projections with sharply-pointed tips; cucullus weak, apically truncate, inner surface covered entirely with fine setae but lacking a corona. Aedeagus tubular, 5 × as long as wide, with a spinulose patch on distal right side and an elongate extension onto base of vesica on left. Vesica as long as aedeagus and 0.5 × as wide as long, oriented 90° to aedeagus, anvil-shaped with short blunt projection dorsad and leftward and long tapering distal portion extending ventrad and rightward; with two subapical diverticula, larger diverticulum on posteroventral aspect ovoid with dense covering of short cornuti oriented basad, smaller diverticulum on ventral portion rounded, without cornuti. **Female genitalia** – Ovipositor lobes pad-like, covered densely with long thin setae. Abdominal segment VIII 3 × as wide as long; anterior and posterior apophyses nearly equal in length, 0.67 × as long as segment VIII. Ostium bursae moderately sclerotized similar to adjacent posterior ductus bursae, nearly as wide as segment VIII. Ductus bursae 6.8 × as long as segment VIII, divided into two segments of equal length; posterior segment trapezoidal, evenly sclerotized, tapering anteriorly to a narrow waist 1/3 × as wide as ostium bursae; anterior segment cylindrical, strongly sclerotized except for thin ventral slit along entire length, anterior portion with long extensions onto dorsal and ventral corpus bursae. Corpus bursae membranous, unisaccate and elongate, 1.75 × as long as ductus bursae and 0.4 × as wide as long; larger anterior portion ovoid, widest anteriorly, bearing five small rugose ovoid signa, two on mid ventral surface and three on dorsal and anterior end; smaller posterior end with broadly conical projection toward left with ductus seminalis at anterior apex.

**Figures 38–44.**
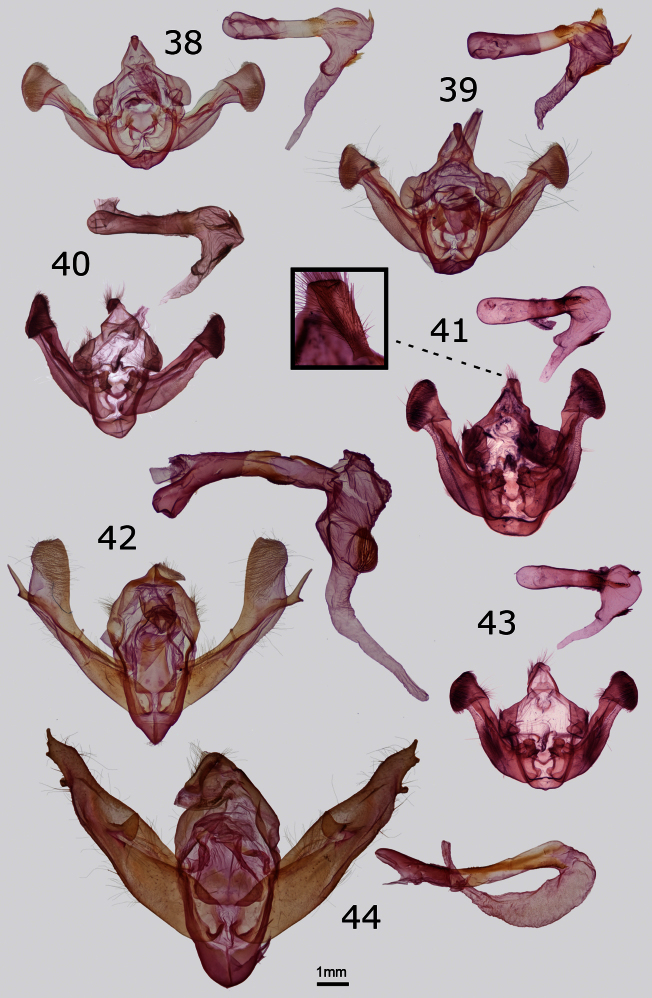
Male genitalia of Noctuidae. Ventral aspect of aedeagus is shown. Distal uncus of Fig. 41 is inset. **38** *Resapamea diluvius* Crabo, paratype, USA, WA, Grant Co., Potholes **39** *Resapamea passer* (Guenée), USA, WA, Douglas Co., 5 km ESE of Orondo **40** *Resapamea angelika* Crabo, holotype, USA, NV, Elko Co., Angel Lake **41***Resapamea mammuthus* Crabo, holotype, Canada, YT, Old Crow **42** *Resapamea hedeni* (Graeser), Russia, Magadan Oblast, Tenkinsky District, Stokovyi **43** *Fishia nigrescens* Hammond & Crabo, paratype, USA, OR, Klamath Co., 6 mi. SE of Klamath Falls **44** *Xestia perquiritata orca* Crabo & Hammond, paratype, USA, WA, Clallam Co., Neah Bay.

**Figures 45–52.**
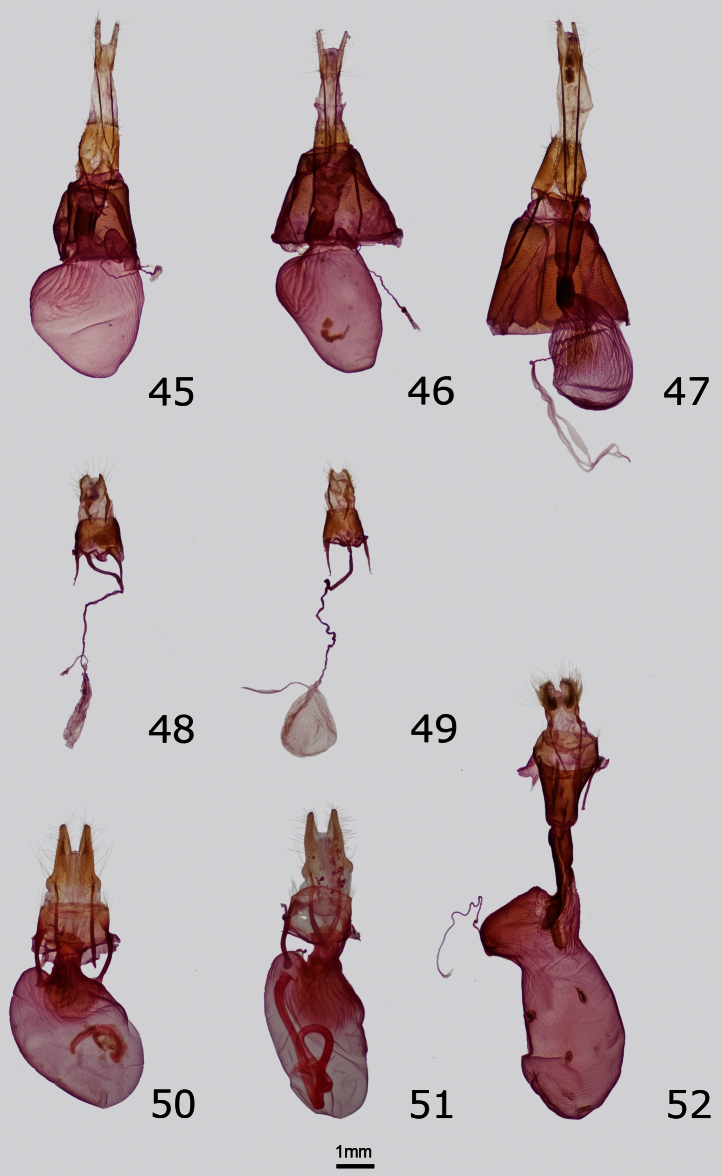
Female genitalia of Erebidae and Noctuidae. Ventral aspect. **45** *Drasteria parallela* Crabo & Mustelin, USA, CA, Siskiyou Co., Deadfall Meadow **46** *Drasteria convergens* Mustelin, USA, CA, Mono Co., Lee Vining **47** *Drasteria divergens* (Behr), USA, Baker Co., Burnt River Canyon **48** *Eudryas brevipennis bonneville* Shepard & Crabo, paratype, USA, ID, Twin Falls Co., Kimberly **49** *Eudryas unio* (Hübner), USA, WI, St. Croix Co., S18 Springfield Township **50** *Resapamea diluvius* Crabo, paratype, USA, WA, Adams Co., Washtucna **51** *Resapamea passer* (Guenée), USA, WA, Douglas County, 3 mi. ESE of Orondo **52** *Fishia nigrescens* Hammond & Crabo, paratype, USA, OR, Jefferson Co., Warm Springs.

##### Distribution and biology.

*Fishia nigrescens* occurs in central and eastern Oregon, Nevada, eastern California, and Arizona. Its flight season is late fall, usually during October. The habitat is sage steppe, often in open juniper forest in Oregon. A specimen of this species was reared from rabbitbrush (*Ericameria nauseosa* (Pallas ex Pursh) G.L. Nesom & Baird) (Asteraceae) in Oregon. The larva was collected in May, pupated in June, and emerged in late September of the same year. It was described as green with a white lateral band.

##### Remarks.

*Fishia yosemitae* was described as *Cucullia yosemitae* Grote. The extant type at the AMNH and those of its synonyms described from western North America, *Fishia exhilarata* Smith and *Fishia betsia* Smith, were examined from photographs to exclude the possibility that one of them could be the correct name for *Fishia nigrescens*.

Specimens of *Fishia nigrescens* from Oregon are darker than those from Nevada and eastern California, whereas those from Arizona are slightly lighter gray.

### Tribe Noctuini Latreille, 1809

#### Xestia perquiritata orca

Crabo & Hammond ssp. n.

http://species-id.net/wiki/Xestia_perquiritata_orca

Figs 30 , 44

##### Type Material.

**Holotype** Male. [USA], Oregon, [Lincoln County], Newport, 25.VII.1961. CNC. **Paratypes** 5 Males, 1 Female. **USA**. **Oregon**. Lincoln County: Newport, 20.VIII.1968, K. J. Goeden collector, black-lite trap (1 female); [same locality and collector], 1.VIII.1970 (2 males). 5 mi. S. Newport 4 VIII 1968, Ore. Dept. Agric. (2 males); [same locality], 30 VII 1970, Brown and Goeden leg. (1 male). **Washington**. Clallam County: Neah Bay, 14.VIII.1961, R. E. Miller (1 male); Grays Harbor County: 3 mi. N Copalis Beach, 11.VIII.2010, MV, Gary L. Peters (1 male). WFBM, GPC, LGCC, ODA, OSAC.

##### Etymology.

The subspecies name is derived from *Orcinus orca*, the killer whale, and is a noun in apposition. It refers to the moth’s Pacific Coast distribution, large size, and nearly black and white forewing.

##### Diagnosis.

Subspecies *orca* is larger than other populations of *Xestia perquiritata* (Morrison). It is the only subspecies in which the wing is uniform black with unmarked white filling of the lines and spots. The western mountain subspecies *Xestia perquiritata partita* (McDunnough) (Fig. 31) has occasional melanic specimens. These are smaller than *Xestia perquiritata orca* (forewing length 16–20 mm for subspecies *partita*; 21–23 mm for *Xestia perquiritata orca*) and differ in having light gray forewing lines and spots as well as darker gray scales in the central reniform spot. These markings are pure white or cream in subspecies *orca*.

The valve of the male genitalia of *Xestia perquiritata orca* is more massive than that of subspecies *partita*, reflecting the larger size of the moth. The female genitalia of *Xestia perquiritata orca* were not examined but are unlikely to differ significantly from those of other *Xestia perquiritata* populations illustrated in Lafontaine (1998).

##### Description.

**Head** – Antenna of male beadlike with short anterior and posterior fasciculations. Antenna of female filiform. Scape with black and white scales, predominantly black dorsally and white ventrally. Eye rounded, smooth. Labial palp covered in gray and blackish-gray scales, scales short on sides and lengthening to a ventral fringe on first two segments. Frons smooth, covered by thin scales that are white centrally and black on sides of head. Top of head covered by thin black scales with scattered white scales in midline and on posterior aspect. **Thorax** – Vestiture of weakly spatulate white-tipped black and scattered white scales, appearing black. Prothoracic collar black with pale-gray edge. Tegula covered by black scales. Legs covered by dark-gray scales with a white ring on each distal segment; medial tibia with a loose row of spiniform setae; ventral tarsal segments with three rows of spiniform setae. **Wings** – Forewing length of males 21–23 mm; females 22 mm. Forewing ground color nearly even brownish black; darkest black in basal area posterior to cubital vein, in fold in medial area, opposite cell and in fold in subterminal area, and between veins in subterminal area; palest whitish gray anterior to cubital vein across antemedial area; slightly mottled with smudged darker veins in terminal area. Basal, antemedial, and postmedial lines similar, black, double, with nearly white pale-cream filling; mostly evident as filling because of dark ground. Basal line pale filling fused to adjacent pale anterior antemedial area. Antemedial line strongly excurved with apex at base of claviform spot, drawn toward base on veins. Medial line absent. Postmedial line toothed on veins; sharply displaced toward base on costa to top of reniform spot, nearly straight from below costa to M3, then angled toward base and slightly curved to meet posterior margin at nearly a right angle. Subterminal line pale gray near costa and evident elsewhere as a transition between black in subterminal area and slightly lighter terminal area. Terminal line a series of black spots between veins. Orbicular and reniform spots completely filled with light cream, often fused. Orbicular spot irregularly ovoid. Reniform spot sideways heart shaped with a deep lateral indentation. Fringe dark gray, weakly scalloped with black. Dorsal hindwing sooty dark gray, faintly brown tinted in some specimens, with faintly darker thin discal spot, postmedial line, veins, and thin terminal line. Hindwing fringe light gray with darker base. **Abdomen** – Blackish gray, slightly lighter than thorax. **Male genitalia** – Uncus strap-like, dorso-ventrally flattened with a spatulate tip. Tegumen with weak penicillus lobes. Juxta broad, 1.8 × as wide as long, with straight transverse ventral margin and bilobed dorsal margin with a slight notch at base of aedeagus. Valve elongate, 4.5 × as long as wide, with slightly undulating costal and ventral margins, tapering to a point distal point with additional small projection from subapical ventral margin and an elongate pollex typical of genus *Xestia* (Lafontaine 1998). Sacculus moderate, expanded to reach costal margin at base of valve and tapering distally to end at mid-valve. Clasper shark-fin shaped, oriented slightly mesially at origin and curved nearly 90° laterally to project dorsolaterally with tip slightly dorsal to costa. Digitus weak, broadly triangular. Apex of valve as described above without an expanded cucullus or corona. Aedeagus long and tubular, 8 × as long as wide, gradually widening from mid-shaft to apex, bearing patches of fine spinules on left side of mid-portion and on right side of apex. Vesica a simple membranous tube, 0.8 × as long as aedeagus and 0.3 × as wide as long; basilar vesica recurved 180° dorsad and rightward so that mid-vesica projects toward base of aedeagus and distal portion curved additional 45° leftward to overlap aedeagus. **Female genitalia** – Not examined.

##### Distribution and biology.

This moth is restricted to the immediate Pacific Coast of Oregon and Washington, usually within several hundred meters of the shore. It has been collected from the vicinity of Newport on the central Oregon coast north to Neah Bay on the northwest tip of the Olympic Peninsula in Washington. Subspecies *Xestia perquiritata orca* flies during late July and August. The food plant is unknown, but other subspecies of *Xestia perquiritata* have been reared from conifers in the family Pinaceae, particularly firs (*Abies* spp.) and spruces (*Picea* spp.) (Lafontaine 1998). Based on the composition of the forests where *Xestia perquiritata orca* occurs the most likely food plant is Sitka spruce (*Picea sitchensis* (Bong.) Carr.), although grand fir (*Abies grandis* (Dougl.) Forbes) is possible.

##### Remarks.

The genus *Xestia* was revised for North America by Lafontaine (1998). He considered a specimen in the CNC from Newport, Oregon to be a melanic *Xestia perquiritata partita* (McDunnough), the western North American subspecies of this boreal and montane moth. At the time, the coast population was only known from a few specimens from this locality and only one of these was at the CNC. It is now apparent that distinct coastal populations of *Xestia perquiritata* have a range that extends at least as far north as the Olympic Peninsula and are nearly uniform in appearance. This subspecies might have a wider distribution on the coasts of northwestern California, British Columbia, and Alaska.

**Figure 53.**
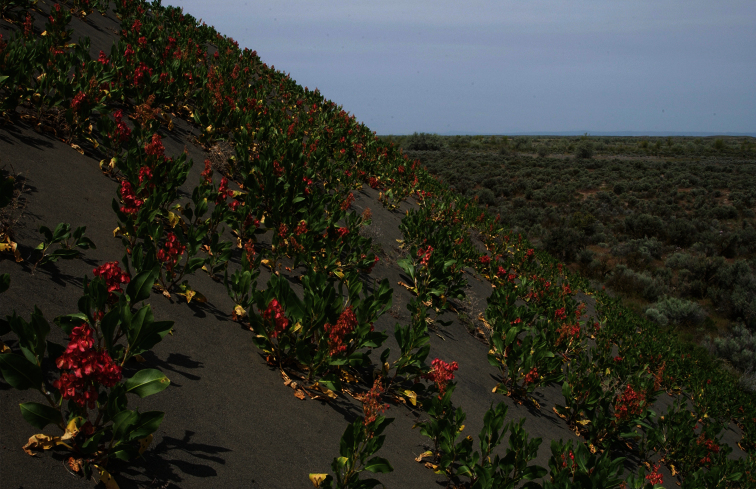
Dune habitat of *Resapamea diluvius* at Potholes, Grant County, Washington, showing the likely foodplant *Rumex venosus* in flower during May.

## Discussion

The taxonomic changes and new taxa descriptions in this paper are part of an ongoing effort to understand fully the macromoth fauna of the PNW. Once published, these changes will be added to the website Pacific Northwest Moths (http://pnwmoths.biol.wwu.edu/ ).

Although the focus of this work is regional, the genera *Resapamea* and *Hydraecia* in the tribe Apameini of the Noctuidae are partially revised herein. This piecemeal approach may be less optimal than an all-encompassing generic revision, however, there are at present barriers that preclude satisfactory completion of such a study in both genera. *Resapamea* and thespecies in the *Hydraecia obliqua* species-group are similar in that structural differences between species are slight (e. g., Figs 38–41). Therefore, other distinguishing features such as differences in biology or DNA sequences, like barcodes, assume greater importance for defining species boundaries. The most readily available DNA sequencing techniques require recently collected material, preferably less than ten years old. Most North American museum material is significantly older than this and fresher material is not currently available for these species from areas that are likely to be critical to solving the remaining quandaries. Collectors are encouraged to seek out and submit specimens for DNA sequencing of *Hydraecia* from the east slope of the Cascades in Oregon and the Southwest, and *Resapamea innota* and *Resapamea enargia* from any part of the western United States. Any *Hydraecia* specimens from central British Columbia or near the border of Alberta and British Columbia might shed light on the relationship of *Hydraecia intermedia* to *Hydraecia obliqua*.

## Supplementary Material

XML Treatment for Cycnia oregonensis tristis

XML Treatment for Chytolita morbidalis

XML Treatment for Drasteria parallela

XML Treatment for Eudryas brevipennis bonneville

XML Treatment for Resapamea diluvius

XML Treatment for Resapamea angelika

XML Treatment for Resapamea mammuthus

XML Treatment for Hydraecia obliqua

XML Treatment for Hydraecia medialis

XML Treatment for Fishia nigrescens

XML Treatment for Xestia perquiritata orca
